# ATF6 aggravates acinar cell apoptosis and injury by regulating p53/AIFM2 transcription in Severe Acute Pancreatitis

**DOI:** 10.7150/thno.46934

**Published:** 2020-07-09

**Authors:** Jie-Hui Tan, Rong-Chang Cao, Lei Zhou, Zhi-Tao Zhou, Huo-Ji Chen, Jia Xu, Xue-Mei Chen, Yang-Chen Jin, Jia-Yu Lin, Jun-Ling Zeng, Shu-Ji Li, Min Luo, Guo-Dong Hu, Xiao-Bing Yang, Jin Jin, Guo-Wei Zhang

**Affiliations:** 1 Division of Hepatobiliopancreatic Surgery, Department of General Surgery, Nanfang Hospital, Southern Medical University, Guangzhou, China.; 2 Department of the Electronic Microscope Room, Central Laboratory, Southern Medical University, Guangzhou, China.; 3 School of Traditional Chinese Medicine, Southern Medical University, Guangzhou, China.; 4 Department of Pathophysiology, Southern Medical University, Guangzhou, China.; 5 Department of Occupational Health and Medicine, Guangdong Provincial Key Laboratory of Tropical Disease Research, School of Public Health, Southern Medical University, Guangzhou, China.; 6 The First Clinical Medical College, Southern Medical University, Guangzhou, China.; 7 Laboratory Animal Research Center of Nanfang Hospital, Southern Medical University, Guangzhou, China.; 8 Guangdong-Hong Kong-Macao Greater Bay Area Center for Brain Science and Brain-Inspired Intelligence, Southern Medical University, Guangzhou, China.; 9 Department of Laboratory Medicine, Nanfang Hospital, Southern Medical University, Guangzhou, China.; 10 Department of Respiratory and Crit Care Medicine, Nanfang Hospital, Southern Medical University, Guangzhou, China.; 11 Division of Nephrology, Nanfang Hospital, Southern Medical University, National Clinical Research Center for Kidney Disease, State Key Laboratory of Organ Failure Research, Guangdong Institute, Guangzhou, China.; 12 Department of Gynaecology and Obstetrics, Nanfang Hospital, Southern Medical University, Guangzhou, China.

**Keywords:** severe acute pancreatitis, ER stress, AIFM2, ATF6, p53, apoptosis

## Abstract

**Background:** There is no curative therapy for severe acute pancreatitis (SAP) due to poor understanding of its molecular mechanisms. Endoplasmic reticulum (ER) stress is involved in SAP and increased expression of ATF6 has been detected in SAP patients. Here, we aimed to investigate the role of ATF6 in a preclinical SAP mouse model and characterize its regulatory mechanism.

**Methods:** Pancreatic tissues of healthy and SAP patients were collected during surgery. Humanized PRSS1 transgenic mice were treated with caerulein to mimic the SAP development, which was crossed to an ATF6 knockout mouse line, and pancreatic tissues from the resulting pups were screened by proteomics. Adenovirus-mediated delivery to the pancreas of SAP mice was used for shRNA-based knockdown or overexpression. The potential functions and mechanisms of ATF6 were clarified by immunofluorescence, immunoelectron microscopy, Western blotting, qRT-PCR, ChIP-qPCR and luciferase reporter assay.

**Results:** Increased expression of ATF6 was associated with elevated apoptosis, ER and mitochondrial disorder in pancreatic tissues from SAP patients and PRSS1 mice. Knockout of ATF6 in SAP mice attenuated acinar injury, apoptosis and ER disorder. AIFM2, known as a p53 target gene, was identified as a downstream regulatory partner of ATF6, whose expression was increased in SAP. Functionally, AIFM2 could reestablish the pathological disorder in SAP tissues in the absence of ATF6. p53 expression was also increased in SAP mice, which was downregulated by ATF6 knockout. p53 knockout significantly suppressed acinar apoptosis and injury in SAP model. Mechanistically, ATF6 promoted AIFM2 transcription by binding to p53 and AIFM2 promoters.

**Conclusion:** These results reveal that ATF6/p53/AIFM2 pathway plays a critical role in acinar apoptosis during SAP progression, highlighting novel therapeutic target molecules for SAP.

## Introduction

Acute pancreatitis (AP) with persistent single or multiple organ failure (MOF) and/or local pancreatic complications is characterized as severe acute pancreatitis (SAP) 1,2. SAP patients with persistent organ failure have a high risk of early mortality within the first 2 weeks after diagnosis, with an overall mortality of over 40%. However, none of the pharmacological treatment studies decrease short-term mortality (in-hospital mortality or mortality within six months) in people with SAP 3. Therefore, the treatment of SAP patients with organ failure currently remains largely supportive 4,5 due to a lack of understanding about SAP-specific regulatory mechanisms.

Premature trypsinogen activation, ER stress and apoptosis are the main contributors to AP pathogenesis 6. In the past two decades, numerous studies using experimental models have suggested that prematurely activated intra-acinar trypsinogen, coded by PRSS1, is sufficient to initiate AP 7. Furthermore, increased trypsin activity, can cause ER stress, is the key mechanism that makes mice more susceptible to pancreatitis 8. In human, this notion was strongly supported by the observation that gain-of-function mutant PRSS1 genes are associated with hereditary pancreatitis 9. In view of this, we have constructed the humanized PRSS1 transgenic mouse for next study.

The ER is critical for the proper folding, maturation and secretion of transmembrane and secreted proteins 10. Activating transcription factor 6 (ATF6) is a key sensor protein on the ER membrane orchestrating the unfolded protein response (UPR), which helps the cell adapt to ER stress 11. ATF6-induced disturbance of fatty acid metabolism caused mitochondrial dysfunction, which led to apoptosis during the development of tubulointerstitial fibrosis in patients with chronic kidney disease 12. ATF6, but not other ER stress sensors, was specifically activated during apoptosis in myoblasts 13. ATF6-mediated signaling pathway of the UPR was involved in nonsteroidal anti-inflammatory drug-induced apoptosis 14. Moreover, ATF6 promoted inflammation during chronic pancreatitis (CP) progression 15. However, whether and how ATF6 participates in ER stress-mediated acinar apoptosis during SAP progression remains undiscovered.

In present study, PRSS1^Tg^ mice were treated with caerulein to establish a preclinical trypsin-induced SAP model, and then analyzed by crossing to ATF6 knockout mice, in addition to using a proteomics-based approach for identifying regulatory partners of ATF6. Apoptosis-inducing factor mitochondria-associated 2 (AIFM2), a p53-inducing protein localized in the mitochondrial outer membrane or freely distributed in the cytoplasm 16,17, as an important downstream regulatory partner of ATF6. AIFM2 protein expression was upregulated when ATF6 was activated and augmented apoptotic signaling pathway activity in response to ER stress. Moreover, AIFM2 was downregulated upon ATF6 or p53 knockout, resulting in decreased apoptosis and inflammation. Consistent with the above regulatory relationship, the structures of the ER, mitochondria and nucleus changed noticeably upon SAP induction. These results point to the ATF6-p53-AIFM2 pathway as a critical factor in ER-induced apoptosis during SAP development, and this knowledge will contribute to the enhancement of clinical management strategies.

## Materials and Methods

### Human pancreatic tissues and study approval

Human pancreatic tissues were collected from 6 SAP patients (mean age = 48.33 ± 4.61) and 12 patients with benign pancreatic tumours or peritumoural normal pancreatic tissues (mean age = 38.50 ± 11.54) as controls. All human samples and matching clinical data were obtained from Nanfang Hospital, Southern Medical University, between 2014 and 2019 (Supplemental Table S1). Written consent was obtained from each patient before the study, which were approved by the Ethics Committee of the Southern Medical University.

### Mouse models

The generation of preclinical PRSS1 (GenBank Accession Number: NM_002769.4) transgenic (PRSS1^Tg^) mice and ATF6 (NCBI Reference Sequence: NM_001081304; Ensembl: ENSMUSG00000026663) knockout mice has been described previously 15. TRp53 (NCBI reference sequence: NM_011640.3; Ensembl: ENSMUSG00000059552) knockout mice were purchased from Cyagen (Guangzhou, China). The tails of pups were genotyped by PCR followed by sequence analysis.

Sex- and age-matched mice (20-25g) were used for assays. Single transgenic littermates subjected to the same treatment were used as controls. All mouse colonies were maintained in a specific pathogen-free barrier facility at the laboratory animal center of Nanfang Hospital, Southern Medical University. Experimental procedures were carried out in strict accordance with the Guide for the Care and Use of Laboratory Animals of the National Institutes of Health and were approved by the Institutional Animal Care and Use Committee of Southern Medical University.

### ATF6- and shAIFM2-expressing adenovirus

To investigate the effect of ATF6 on apoptosis and inflammation, the pancreases of PRSS1^Tg^ mice were infected with adenoviral vectors harboring full-length ATF6 for ATF6 overexpression or a scrambled adRNA for the negative control, respectively. Similarly, to investigate the effect of AIFM2 on apoptosis and inflammation, the pancreases of PRSS1^Tg^ mice were infected with adenoviral vectors harboring full-length AIFM2 for AIFM2 overexpression, shAIFM2 fragments for AIFM2 silencing or a scrambled shRNA for the negative control. All adenoviral vectors were purchased from VectorBuilder Inc. (Guangzhou, China). The sequences of the primers and the adenoviruses used are listed in Supplemental Table S2.

### SAP induction and treatment

To establish a SAP model, PRSS1^Tg^ mice were intraperitoneally injected with 15 μg/mL caerulein dissolved in phosphate-buffered saline (PBS) at 50 μg/kg every hour for a total of 8 injections and sacrificed 24 hours later. For ATF6 or AIFM2 overexpression and AIFM2 inhibition, the PRSS1^Tg^ mice were injected with adenovirus three days prior to caerulein injection. Mice were intraperitoneally injected with 2.2 mg/kg pifithrin-α (PFT-α, a p53 inhibitor) each day before the caerulein injection to inhibit p53 expression.

### Proteomics

PRSS1^Tg^ mice were treated with caerulein (group A) or saline (group B) and PRSS1^Tg^/ATF6^-/-^ mice were treated with caerulein (group C). Caerulein or saline treatment was performed for 8 hours and mice were sacrificed 24 hours later.

For proteomic analysis, tandem mass spectrometry data was collected for peptide identification through automated data-dependent acquisition (DDA). Using the DDA method, mass information on intact peptides in a full-scan mass spectrum was used to determine which subset of mass signals should be targeted for further acquisition of fragmentation spectra to identify peptide sequences.

Protein extraction, quality control of the extraction process and proteolysis were conducted. Fractions from each sample were analyzed by both DDA and data-independent acquisition (DIA) methods on a Q Exactive HF X mass spectrometer (Thermo Fisher Scientific) coupled to an Ultimate 3000 RSLC Nano system (Thermo Fisher Scientific). DDA data were identified using the Andromeda search engine within MaxQuant, and the identification results were used in Spectronaut™ for spectral library construction. For large-scale DIA data, the constructed spectral library information was used in Spectronaut™ to complete deconvolution and extraction, and the mProphet algorithm was used to complete analytical quality control, thus generating a large number of reliable quantitative results. This pipeline was also used to perform Gene Ontology (GO), Clusters of Orthologous Groups (COG), pathway functional annotation and time series analyses.

### Transmission electron microscopy (TEM) and immunoelectron microscopy (IEM)

Ultrastructural examination was performed using a transmission electron microscope according to a previously described protocol 18. Human pancreatic tissues (1 mm^3^) and mice tissues (1 mm^3^) from the pancreas, lung, liver, kidney, duodenum, heart, and spleen were fixed with 2.5% glutaraldehyde at room temperature for 1 hour and then at 4°C overnight. After fixation, the samples were rinsed in PBS three times for 10 min each.

For immunogold staining, the experimental methods has been adjusted, referring to the previous literatures 19,20. Mice pancreatic tissues (1 mm^3^) were fixed with a mixture of 4% paraformaldehyde and 0.1% glutaraldehyde dissolved in 0.1 mol/L phosphate buffer (PB) at 4°C overnight. Then, the specimens in PBS were sliced into 50-μm sections on a vibrating microtome (Lecia VT1200S). The sections were then processed following standard procedures in Supplemental Methods. The resin-embedded sections were cut into ultrathin sections (70-80 nm thickness) and were collected on nickel grids (Nisshin EM). Finally, the sections were analyzed with an electron microscope (Hitachi H-7500, Japan) operated at 60 kV.

### Quantitative real-time PCR (qRT-PCR)

qRT-PCR was performed to measure mRNA expression in pancreatic tissues. The sequences of the primers used are listed in Supplemental Table S2. Please see the Supplementary Methods for more details.

### Western blotting

Total protein was extracted from the mice pancreatic tissues. Primary antibodies used in this study include anti-ATF6 (Abcam; diluted 1: 500), anti-p53 (Abcam; diluted 1: 500), anti-AIFM2 (Biorbyt; diluted 1: 500), and anti-GAPDH (Abcam; diluted 1:1000). The protein abundance was evaluated by immunoblotting with at least three biological replicates. Please refer to the Supplemental Methods for more details.

### Immunohistochemistry and Immunofluorescence assay

H&E staining and immunohistochemistry analysis of pancreatic tissue slides for ATF6, p53, AIFM2, Caspase-3, PARP and MPO were performed according to a previously described protocol 21. To visualize ATF6, p53 and AIFM2 colocalization in pancreatic tissue, immunofluorescence assay was also performed. Experimental details can be obtained by the Supplemental Methods.

### Transferase-mediated d-UTP nick-end-labeling (TUNEL) assay

Terminal deoxynucleotidyl TUNEL was performed to detect apoptosis. For more details, please refer to the Supplemental Methods.

### Enzyme-Linked Immunosorbent Assay (ELISA)

Supernatants were collected and stored at -80℃ after removal of serum by centrifugation. The protein concentration of IL-1β, IL-6, and TNF-α in the serum were detected using specific ELISA kits (ROCHE COBAS8000 E602, Switzerland) according to the manufacturer's instructions.

### Chromatin immunoprecipitation (ChIP) assay

Chromatin immunoprecipitation (ChIP) assays were performed using the Pierce Magnetic ChIP Kit (Thermo Fisher Scientific) according to the manufacturer's instructions. Briefly, pancreatic acinar cells were collected from PRSS1^Tg^ mice and treated with caerulein or untreated. Cells were cross-linked with 1% formaldehyde for 10 minutes at room temperature, followed by nucleus separation and sonication in lysis buffer. After centrifugation, the supernatant was separated and used for western blotting to detect the expression of ATF6. ChIP grade anti-ATF6 antibody (Abcam) and protein G magnetic beads were used for immunoprecipitation of chromatin. Normal rabbit IgG served as negative control. Finally, PCR analysis using the PCR Kit (Promega) was performed on purified input and immunoprecipitated DNA. The sequences of the primers for promoter region used are listed in Supplemental Table S3.

### Luciferase assay

A series of deletion mutants of p53 promoter (chr11:69578359-69580458) and AIFM2 promoter (chr10:61713263-61715362) regions were respectively generated into promoter luciferase constructs, which were then inserted into the pGL3 vector. Subsequently, ATF6 CDS sequence (1971bp) was cloned into pcDNA3.1 plasmid. 293T cells were grown in 24-well plates and co-transfected with plasmids containing a series of deletion mutants of p53 promoter or AIFM2 promoter and either ATF6 overexpression plasmids or the empty control plasmids using the Lipofectamine 2000 Reagent (Invitrogen). Cellular extracts were measured for luciferase activity using the luciferase reporter assay system at 48h. Each experiment was repeated three times.

### Statistical analysis

Statistical analysis was carried out using GraphPad Prism 8.0. The data are expressed as mean ± standard error of the mean. Significant differences between two groups were analyzed by Student's t-test, and one-way analysis of variance was performed to investigate the differences among more than two groups. Significant differences were defined by a *P* value of < 0.05.

## Results

### Elevated ATF6 expression in AP is associated with increased apoptosis and structural damage to the ER and mitochondria

**Figure 1:**
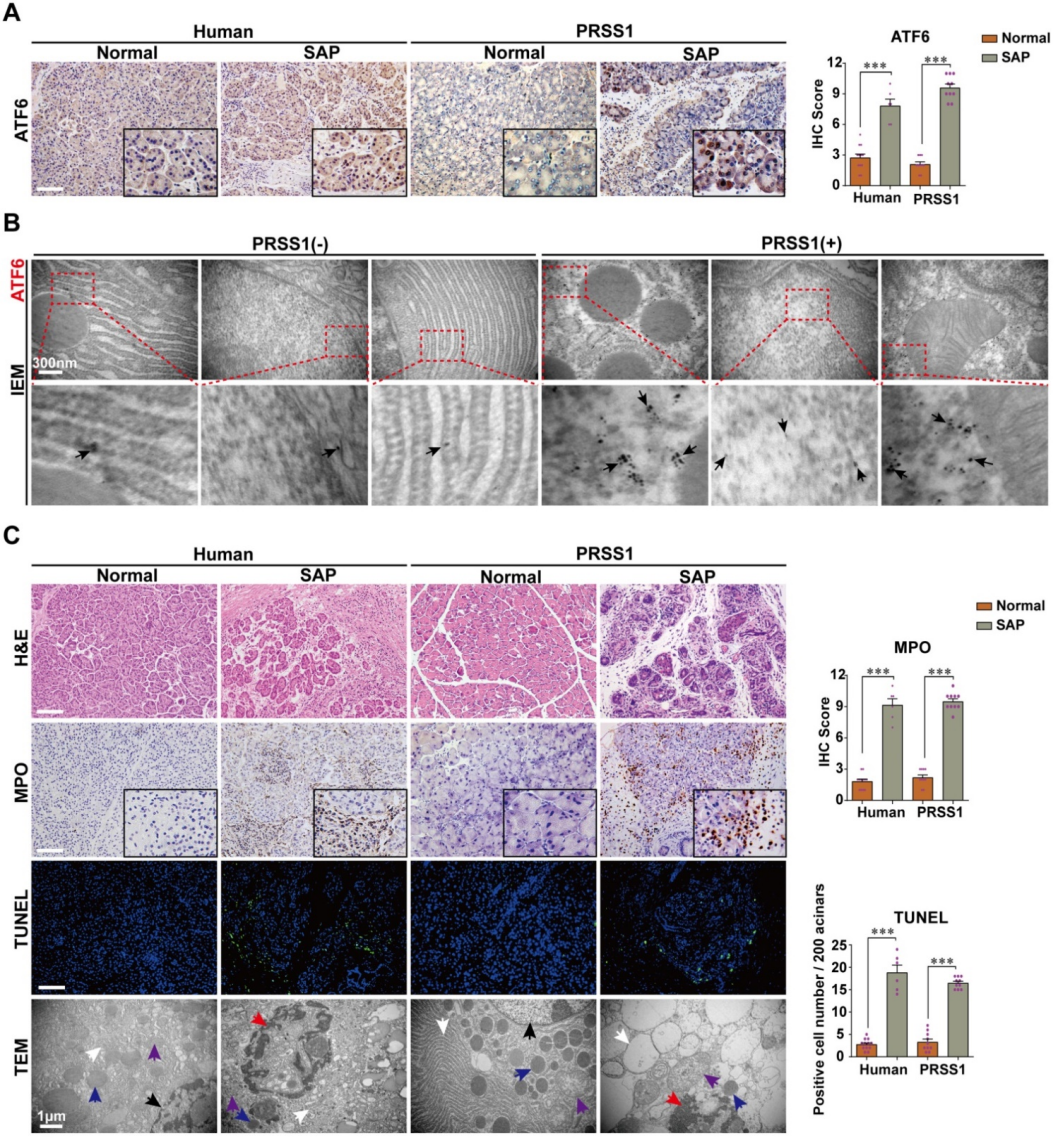
** ATF6 is upregulated in SAP pancreatic tissues from patients and PRSS1 transgenic (PRSS1^Tg^) mice accompanying with elevated apoptosis. (A)** ATF6 expression was assessed by immunohistochemistry. **(B)** The localization and expression of ATF6 were examined by immunoelectron microscopy (IEM); black arrows (↑): representative gold nanoparticles. **(C)** Histological alterations, myeloperoxidase (MPO) activity, acinar cell apoptosis, and microstructural changes in pancreatic tissues from human and PRSS1^Tg^ mice were assessed by hematoxylin and eosin (H&E) staining, immunohistochemistry, terminal deoxynucleotidyl transferase dUTP nick end labeling (TUNEL) assays and transmission electron microscopy (TEM) as indicated. black arrows (↑): cell nuclei; white arrows (↑): endoplasmic reticula; blue arrows (↑): zymogen granules; purple arrows (↑): mitochondria; red arrows (↑): apoptotic bodies. (-), not treated with caerulein; (+), treated with caerulein. The data are presented as the means ± SDs; * p ≤ 0.05, ** p ≤ 0.01, *** p ≤ 0.001. Scale bars = 100 µm.

To elucidate the role that increased ATF6 expression plays in SAP pathogenesis, we first sought to identify the phenotypes associated with elevated levels of this protein in SAP patients or human volunteers (detailed in Supplemental Table S1) and a transgenic mouse model. Immunohistochemistry analysis showed that the expression levels of ATF6 in human and PRSS1^Tg^ mouse SAP samples was highly elevated compared with control samples (Figure 1A). Additionally, immunoelectron microscopy (IEM) analysis showed that large amounts of ATF6 localized mainly in the ER and nucleus in SAP tissues from PRSS1^Tg^ mice, while this was rarely observed in pancreatic tissues from untreated PRSS1^Tg^ mice (Figure 1B). Histological analysis showed that myeloperoxidase (MPO) expression, pancreatic acinar cell deformation and inflammatory cell infiltration were markedly increased in human and mouse SAP tissues compared with control tissues (Figure 1C). Ultrastructural analysis by transmission electron microscopy (TEM) revealed extensively irregular and dilated ERs, mitochondrial swelling, nuclear fragmentation and apoptotic bodies were observed in human and PRSS1^Tg^ mouse SAP tissues; however, no obvious abnormal alterations were observed in normal acinar cells (Figure 1C). An increase in the number of apoptotic cells (Figure 1C) and in the expression of the apoptotic cell death markers Caspase-3 and poly ADP-ribose polymerase (PARP) was seen in SAP tissues from PRSS1^Tg^ mice (Figure S1A). These findings suggest a correlation of ATF6 with apoptosis during SAP progression.

### ATF6 promotes acinar cell injury and multiple organ injury in PRSS1^Tg^ SAP model

**Figure 2:**
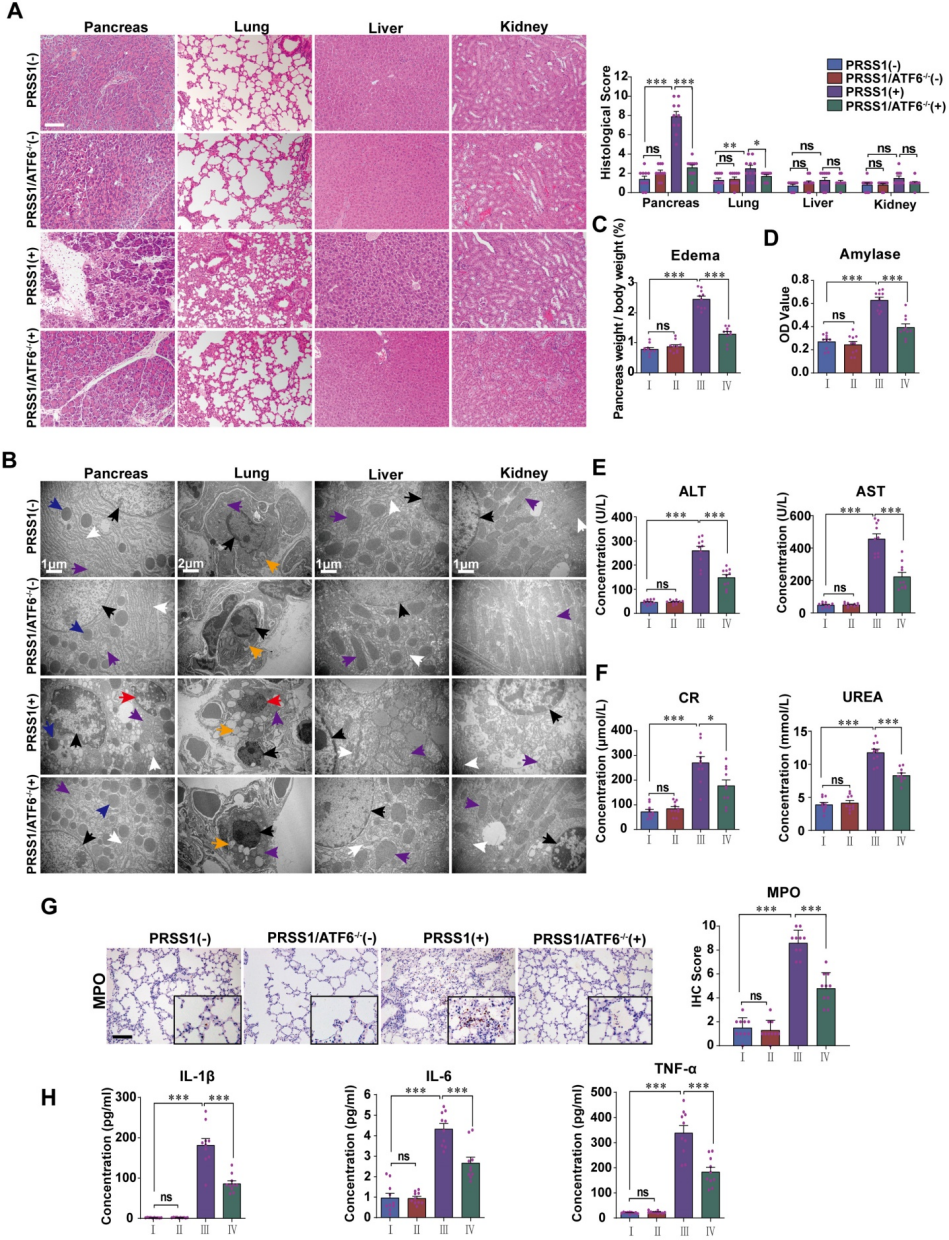
** ATF6 promotes apoptosis, inflammation, and multiple organ injury in the PRSS1^Tg^ SAP mouse model. (A)** Histological and **(B)** microstructural changes in pancreas, lung, liver, and kidney tissues collected from caerulein-treated PRSS1^Tg^ mice and PRSS1^Tg^/ATF6^-/-^ mice were analyzed by H&E staining and TEM, respectively; black arrows (↑): cell nuclei; white arrows (↑): endoplasmic reticula; blue arrows (↑): zymogen granules; purple arrows (↑): mitochondria; red arrows (↑): apoptotic bodies; orange arrows (↑): lamellar bodies. Degree of edema **(C)** and serum amylase level** (D)** in caerulein-treated PRSS1^Tg^ mice and PRSS1^Tg^/ATF6^-/-^ mice. Expression of liver-related ALT and AST **(E)**, kidney-related CR and urea **(F)** in serum from caerulein-treated PRSS1^Tg^ mice and PRSS1^Tg^/ATF6^-/-^ mice. The MPO expression in lung tissues **(G)** from caerulein-treated PRSS1^Tg^ mice and PRSS1^Tg^/ATF6^-/-^ mice.** (H)** The levels of IL-1β, IL-6, and TNF-α in serum were measured by ELISA from caerulein-treated PRSS1^Tg^ mice and PRSS1^Tg^/ATF6^-/-^ mice. (-), not treated with caerulein; (+), treated with caerulein. Group I, PRSS1^Tg^ (-); group II, PRSS1^Tg^/ATF6^-/-^ (-); group III, PRSS1^Tg^ (+); group IV, PRSS1^Tg^/ATF6^-/-^ (+). The data are presented as the means ± SDs; ns, no significant difference; * p ≤ 0.05, ** p ≤ 0.01, *** p ≤ 0.001. Scale bars = 100 µm.

To analyze if increased expression in SAP tissues is indicative of a role for ATF6 during SAP progression, PRSS1^Tg^ mice were crossed with ATF6 knockout (ATF6^-/-^) mice. Caerulein treatment of PRSS1^Tg^ mice ideally mimicked human SAP with multiple organ injury and could serve as a suitable preclinical model for mechanistic research on pancreatic inflammatory diseases. SAP was induced in PRSS1^Tg^ and PRSS1^Tg^/ATF6^-/-^ mice by caerulein treatment, followed by analysis of histology, ultrastructure, and function of pancreas, lung, liver, kidney, duodenum, heart, and spleen. Histological analysis showed that the cell deformation and inflammatory cell infiltration seen in the pancreas and lung upon treatment with caerulein were negated in PRSS1^Tg^/ATF6^-/-^ mice compared to PRSS1^Tg^ mice (Figure 2A). Ultrastructural analysis showed that disorder of ER, swelling of mitochondria, and fragmentation of cell nuclei in pancreatic and liver tissues, swelling and deformation of lamellar bodies in lung tissues, swelling of mitochondria in kidney tissue, destruction of microvilli in duodenum tissue, rupture of myofibrils in heart tissue, and apoptosis of lymphocytes in spleen upon caerulein treatment in PRSS1^Tg^ mice were attenuated in PRSS1^Tg^/ATF6^-/-^ mice, indicating inhibition of caerulein-induced acinar injury in the absence of ATF6 (Figure 2B and Figure S2B). However, no significant changes upon caerulein treatment were observed in the other tissues examined (Figure 2A and Figure S2B). Caerulein-induced increases in the degree of pancreatic edema (Figure 2C and Figure S2A), levels of pancreas-related serum amylase (Figure 2D), liver-related alanine aminotransferase (ALT) and aspartate aminotransferase (AST) (Figure 2E), kidney-related creatinine (CR) and urea (Figure 2F), MPO activity in lung tissue (Figure 2G), heart-related creatine kinase-MB (CK-MB) (Figure S2C), and the inflammatory cytokines IL-1β, IL-6, and TNF-α in serum (Figure 2H) seen in PRSS1^Tg^ mice were negated upon ATF6 knockout. However, no significant changes were observed in the level of heart-related lactate dehydrogenase (LDH) (Figure S2C).

To ensure that the inhibition of caerulein-induced effects in PRSS1^Tg^/ATF6^-/-^ mice was specific to ATF6, complementation was performed in the pancreas using ATF6-expressing adeno-associated virus, with high transfection efficiency. Caerulein-induced cell deformation and inflammatory cell infiltration in pancreatic and lung tissues were reestablished in PRSS1^Tg^/ATF6^-/-^ mice upon restoration of ATF6 expression (Figure S3A). Caerulein-induced ER dilatation, mitochondrial swelling, and peripheral chromatin condensation in pancreatic and liver tissues, lamellar bodies deformation in lung tissues, mitochondrial swelling in kidney tissues, microvilli destruction in duodenum tissue, myofibrils rupture in heart tissues, and lymphocytes apoptosis in spleen tissues were reestablished in PRSS1^Tg^/ATF6^-/-^ mice upon restoration of ATF6 expression (Figure S3B). These effects were not significant in the other tissues examined (Figure S3A, B). Caerulein-induced increases in the degree of pancreatic edema (Figure S3C), levels of serum amylase (Figure S3D), ALT and AST (Figure S3E), CR and urea (Figure S3F), MPO activity in lung tissue (Figure S3G), and the inflammatory cytokines IL-1β, IL-6, and TNF-α in serum (Figure S3I) in PRSS1^Tg^ mice that were inhibited upon ATF6 knockout, were reestablished after ATF6 expression was restored. No significant changes were observed in the CK-MB and LDH levels (Figure S3H) upon restoration of ATF6 expression. Collectively, these results indicate that ATF6 promotes acinar cell injury in PRSS1^Tg^ SAP mouse model.

### AIFM2 is a regulatory partner of ATF6 in SAP

**Figure 3:**
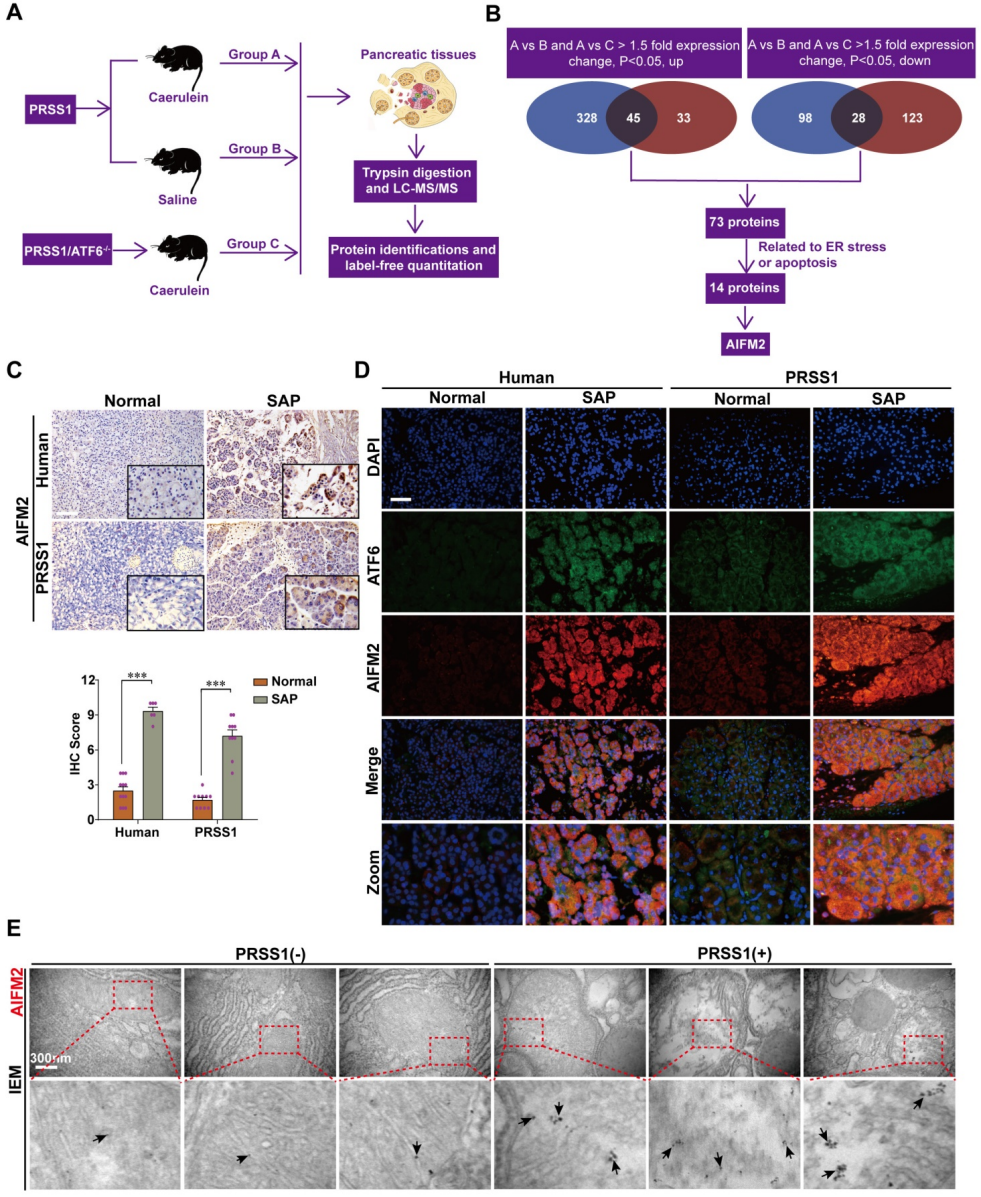
** Proteomic analysis to identify potential regulatory proteins related to SAP progression. (A)** Schematic representation of the protocol followed for proteomic analysis. **(B)** Schematic diagram of proteomic screening process to identify target proteins related to SAP progression. **(C)** Representative images of immunohistochemical staining of AIFM2 in pancreatic tissues from humans and PRSS1^Tg^ mice. **(D)** Immunofluorescence staining to detect the localization of ATF6 and AIFM2 in human and PRSS1^Tg^ mouse pancreatic tissues. **(E)** IEM analysis of AIFM2 localization and expression; black arrows (↑): representative gold nanoparticles. The data are presented as the means ± SDs; ns, no significant difference; * p ≤ 0.05, ** p ≤ 0.01, *** p ≤ 0.001. Scale bars = 100 µm.

To elucidate the mechanism underlying ATF6-mediated promotion of acinar cell injury, pancreatic tissues from PRSS1^Tg^/ATF6^-/-^ mice treated with caerulein were subjected to proteomics analysis, and differentially expressed proteins (DEPs) were identified compared to control animals (Figure 3A). DEPs, with the same changing trends (up or down), from group A versus group B that were also differentially expressed in group A versus group C would be potential ATF6 partners. A total of 73 DEPs were identified. Due to increased apoptosis and structural damage to the ER in SAP, we screened out 14 DEPs, of which functions strongly correlated with apoptosis and ER stress, from the 73 DEPs by performing an analysis with the UniProt database. The data-independent acquisition (DIA) quantification results from the proteomic analysis ultimately identified AIFM2 (an apoptosis-inducing factor (AIF)-homologous mitochondrion-associated protein 2), an inducer of caspase-independent apoptosis 22,23, as a potential downstream regulatory partner of ATF6 (Figure 3B). The upregulated and downregulated proteins were classified by their GO function (Figure S4A-C). A protein-protein interaction (PPI) network of the 14 DEPs was constructed (Figure S4D).

To validate the proteomic identification of AIFM2, we examined its expression in SAP tissues from patients and PRSS1^Tg^ mice by immunohistochemistry and immunofluorescence. Compared with control pancreatic tissues, SAP tissues of humans and PRSS1^Tg^ mice exhibited significantly increased expression of AIFM2 (Figure 3C, D). IEM analysis showed large concentrations of AIFM2 in SAP tissues from PRSS1^Tg^ mice that were localized mainly in mitochondria, but such concentrations were rarely seen in normal pancreatic tissues (Figure 3E). The increased mitochondrial expression of AIFM2 in SAP suggests a role in acinar apoptosis.

**Figure 4:**
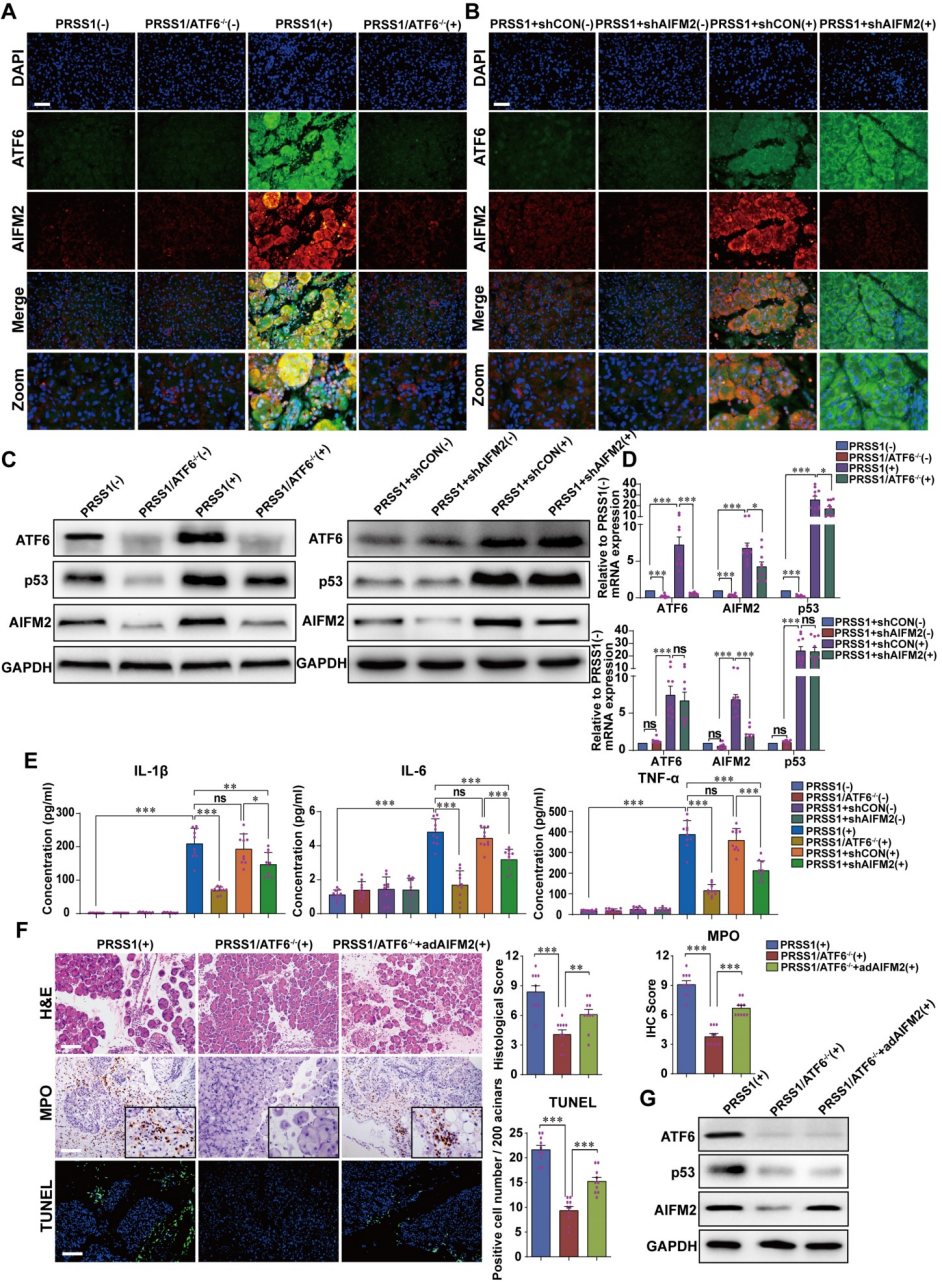
** AIFM2 is regulated by ATF6 in the PRSS1^Tg^ SAP mouse model. (A)** Immunofluorescence staining to examine the colocalization of ATF6 (green fluorescence) and AIFM2 (red fluorescence) in pancreatic tissues from caerulein-treated PRSS1^Tg^ mice and PRSS1^Tg^/ATF6^-/-^ mice. **(B)** shAIFM2 for AIFM2 silencing or control shRNA (shCON) for the negative control were delivered to the pancreas of PRSS1^Tg^ mice. The colocalization of ATF6 (green fluorescence) and AIFM2 (red fluorescence) in pancreatic tissues from PRSS1^Tg^ SAP mice with AIFM2 silencing was examined by immunofluorescence staining. Western blot **(C)** and qRT-PCR **(D)** analyses of ATF6, p53, and AIFM2 expression in pancreatic tissues from caerulein-treated PRSS1^Tg^/ATF6^-/-^ and PRSS1^Tg^ mice with AIFM2 silencing; GAPDH served as the internal loading control. **(E)** Expression of IL-1β, IL-6, and TNF-α in serum were measured by ELISA from caerulein-treated PRSS1^Tg^/ATF6^-/-^ and PRSS1^Tg^ mice with AIFM2 silencing.** (F)** AIFM2-expressing adenovirus (adAIFM2) for AIFM2 overexpression was delivered to the pancreas of PRSS1^Tg^/ATF6^-/-^ mice. Histological alterations, MPO expression, and acinar cell apoptosis in pancreatic tissues from caerulein-treated PRSS1^Tg^ mice, PRSS1^Tg^/ATF6^-/-^ mice, and PRSS1^Tg^/ATF6^-/-^+adAIFM2 mice were measured by H&E staining, immunohistochemistry, and TUNEL, respectively. **(G)** Western blot analyses of ATF6, p53, and AIFM2 expression in pancreatic tissues from caerulein-treated PRSS1^Tg^ mice, PRSS1^Tg^/ATF6^-/-^ mice, and PRSS1^Tg^/ATF6^-/-^+adAIFM2 mice; GAPDH served as the internal loading control. The data are presented as the means ± SDs; ns, no significant difference; * p ≤ 0.05, ** p ≤ 0.01, *** p ≤ 0.001. Scale bars = 100 µm.

To evaluate the regulatory relationship between ATF6 and AIFM2, the expression of AIFM2 upon ATF6 knockout in SAP tissues from PRSS1^Tg^ mice was assessed. Immunofluorescence (Figure 4A, B), western blotting (Figure 4C) and qRT-PCR (Figure 4D) analysis revealed that the increase in mRNA and protein expression of AIFM2 upon SAP induction was attenuated upon knockout of ATF6 in pancreatic tissues of PRSS1^Tg^ mice. However, upon AIFM2 silencing, the increase in the mRNA and protein expression of ATF6 remained unchanged (Figure 4A-D). Moreover, the levels of the inflammatory cytokines IL-1β, IL-6, and TNF-α in serum were significantly increased in caerulein-treated PRSS1^Tg^ mice but significantly reduced in these SAP model mice with ATF6 knockout or AIFM2 silencing (Figure 4E). To further investigate if AIFM2 is regulated by ATF6 in SAP, AIFM2 was overexpressed in the pancreas of caerulein-treated PRSS1^Tg^/ATF6^-/-^ mice using adenovirus-based delivery. The caerulein-induced increases in inflammatory cell infiltration, MPO activity, apoptotic cell number, pancreatic edema, and the level of serum amylase in PRSS1^Tg^ mice that were inhibited upon ATF6 knockout, were partially reestablished after AIFM2 overexpression (Figure 4F and Figure S5A-C). The result of western blotting also revealed that the attenuation in protein expression of AIFM2 upon knockout of ATF6 in pancreatic tissues of caerulein-treated PRSS1^Tg^ mice. However, upon restoration of AIFM2 expression, the increase in the protein expression of ATF6 remained unchanged (Figure 4G). Due to AIFM2 is a p53-inducing protein, p53 expression was also measured by western blotting and qRT-PCR which indicated that the increase in p53 expression was attenuated upon knockout of ATF6 in PRSS1^Tg^ mice SAP tissues, but that remained unchanged when silencing or restoration of AIFM2 expression (Figure 4C, D, G). Collectively, these results suggest a regulatory correlation between AIFM2 and ATF6.

### AIFM2 promotes acinar apoptosis, injury and inflammation in SAP

**Figure 5:**
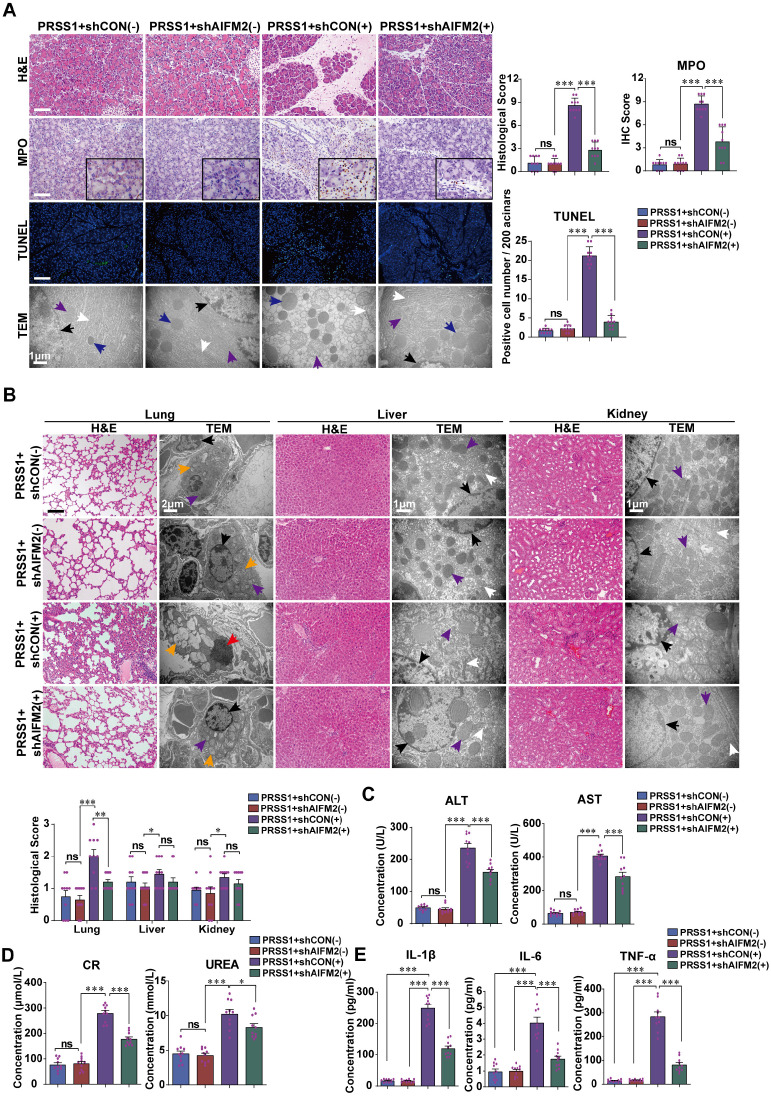
** AIFM2 silencing ameliorates inflammation and multiple organ injury in SAP. (A)** Histological alterations, MPO expression, acinar cell apoptosis, and microstructural changes in pancreatic tissues from PRSS1^Tg^ SAP mice with shAIFM2-mediated AIFM2 silencing were measured by H&E, immunohistochemical staining, TUNEL assays, and TEM, respectively. **(B)** Histological and ultrastructural evaluation of lung, liver, and kidney tissues from PRSS1^Tg^ SAP model mice treated with shAIFM2. Black arrows (↑): cell nuclei; white arrows (↑): endoplasmic reticula; blue arrows (↑): zymogen granules; purple arrows (↑): mitochondria; red arrows (↑): apoptotic bodies; orange arrows (↑): lamellar bodies. The levels of ALT, AST **(C)**, CR and urea **(D)** in serum from PRSS1^Tg^ mice treated with shAIFM2 or shCON. **(E)** Expression of IL-1β, IL-6, and TNF-α in serum were measured by ELISA from PRSS1^Tg^ mice treated with shAIFM2. The data are presented as the means ± SDs; ns, no significant difference; * p ≤ 0.05, ** p ≤ 0.01, *** p ≤ 0.001. Scale bars (H&E, MPO) = 100 µm; scale bars (TUNEL) = 200 µm.

To ascertain the role of AIFM2 in SAP development, we performed shRNA-mediated silencing of AIFM2 through adenoviral transduction in pancreas of PRSS1^Tg^ mice, followed by assessment of pathological changes. The increases in inflammatory cell infiltration, MPO activity, apoptotic cells numbers, ER dilatation, mitochondrial swelling, peripheral chromatin condensation, and levels of inflammatory cytokines observed in PRSS1^Tg^ SAP mice were significantly ameliorated upon AIFM2 silencing (Figure 5A, E). The degree of pancreatic edema, serum amylase level (Figure S6A-C), ALT and AST level (Figure 5C), CR and urea level (Figure 5D) in PRSS1^Tg^ SAP mice that were ameliorated upon AIFM2 silencing. No significant change was observed in the CK-MB and LDH levels (Figure S6F). Consistent with the observations of apoptotic cell numbers, the increase in the expression of Caspase-3 and PARP in PRSS1^Tg^ mouse pancreatic tissues upon SAP induction was markedly negated upon AIFM2 silencing (Figure S6D). Similarly, results of ultrastructural analysis showed that lamellar bodies deformation in lung tissues, mitochondrial swelling in liver and kidney tissues, microvilli destruction in duodenum tissues, myofibrils rupture in heart tissues, and lymphocytes apoptosis in spleen tissues upon SAP induction were markedly negated upon AIFM2 silencing, but no significant differences were observed in the histological analysis of these tissues (Figure 5B and Figure S6E). Collectively, these results indicate that AIFM2 promotes acinar apoptosis during SAP.

### ATF6 regulates the p53-AIFM2 pathway in SAP

**Figure 6:**
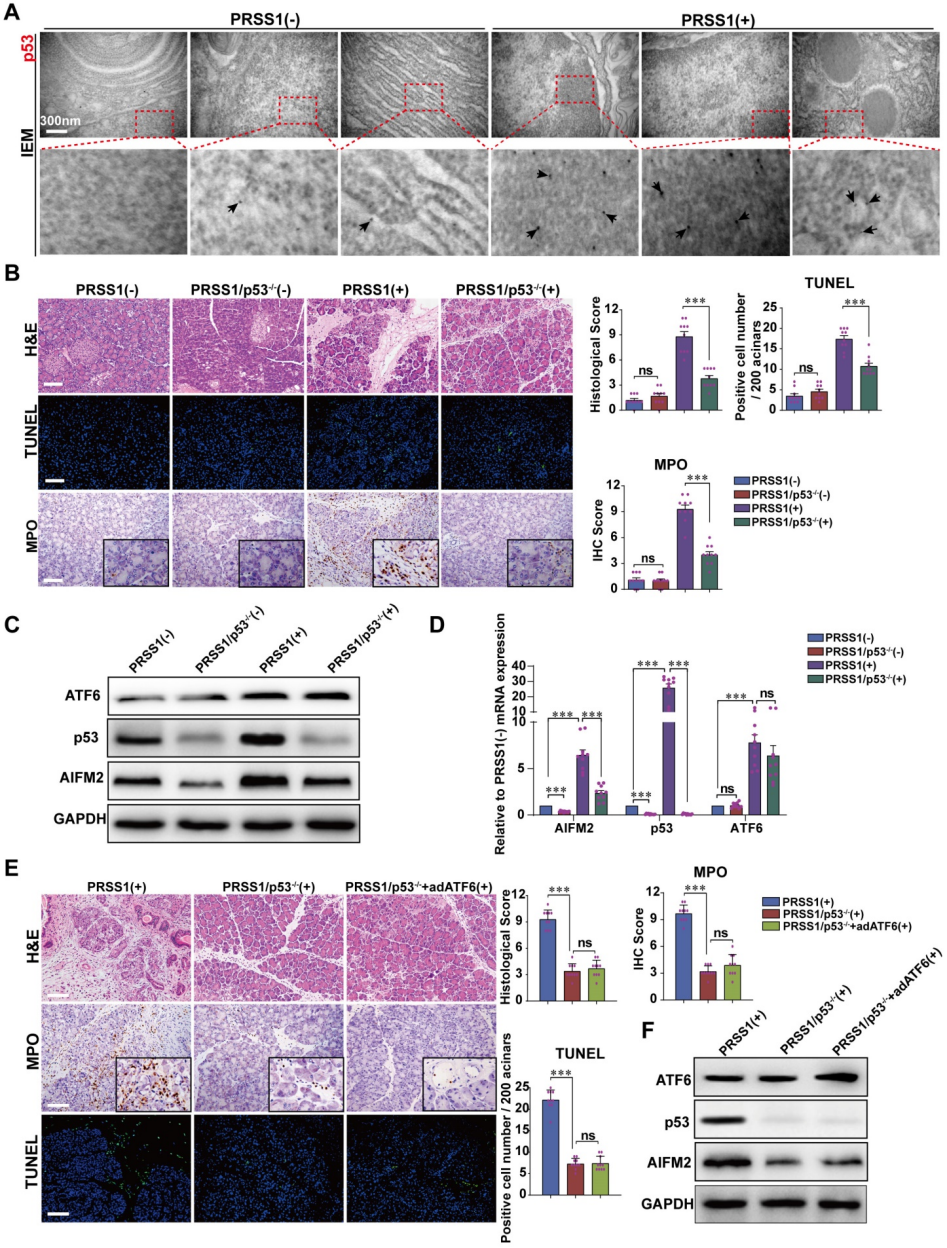
** AIFM2 is regulated by p53 and p53 knockout ameliorates acinar apoptosis and inflammation in SAP. (A)** Microscopic localization and expression of p53 was examined by IEM; black arrows (↑): representative gold nanoparticles. **(B)** Representative images of H&E staining, TUNEL, and MPO activity in caerulein-treated PRSS1^Tg^/p53^-/-^ mice. Western blot **(C)** and qRT-PCR **(D)** analyses of ATF6, AIFM2, and p53 expression in pancreatic tissues from PRSS1^Tg^/p53^-/-^ mice; GAPDH served as the internal loading control. **(E)** Histological alterations, MPO expression, and acinar cell apoptosis in pancreatic tissues from caerulein-treated PRSS1^Tg^ mice, PRSS1^Tg^/p53^-/-^ mice, and PRSS1^Tg^/p53^-/-^+adATF6 mice were assessed by H&E staining, immunohistochemistry, and TUNEL, respectively. **(F)** Western blot analyses of ATF6, p53, and AIFM2 expression in pancreatic tissues from caerulein-treated PRSS1^Tg^ mice, PRSS1^Tg^/p53^-/-^ mice, and PRSS1^Tg^/p53^-/-^+adATF6 mice. The data are presented as the means ± SDs; * p ≤ 0.05, ** p ≤ 0.01, *** p ≤ 0.001. Scale bars = 100 µm.

Given that AIFM2 is a p53-inducible gene, we next ask whether ATF6 regulates AIFM2 and induces apoptosis in SAP by modulating p53 expression. Here, we identified AIFM2 as a regulatory partner of ATF6 in SAP. So, we hypothesize that, during SAP development, ATF6 regulates the p53-AIFM2 pathway via apoptosis. To test this hypothesis, we first examined the expression of p53 in SAP. IEM revealed large concentrations of p53 in PRSS1^Tg^ mouse SAP tissues that were localized mainly in the nucleus and cytoplasm, but such concentrations were rarely seen in normal pancreatic tissues (Figure 6A). Consistent with these results, immunofluorescence analysis revealed that the increased expression of ATF6 and AIFM2 upon SAP induction was associated with increased expression of p53 (Figure S7A, B).

To confirm that p53 is involved in SAP, PRSS1^Tg^ mice were crossed to p53 knockout (p53^-/-^) mice. Compared with caerulein-treated PRSS1^Tg^ mice, caerulein-treated PRSS1^Tg^/p53^-/-^ mice exhibited significantly attenuated pancreatic acinar cell deformation, inflammatory cell infiltration, apoptotic cell numbers, and MPO activity (Figure 6B). Similar results were seen upon p53 inhibition using PFT-α in caerulein-treated PRSS1^Tg^ mice (Figure S8A, B). The reestablishment of caerulein-induced increases in inflammatory cell infiltration, MPO activity, apoptotic cell numbers, ER disorder (Figure S8A), and levels of inflammatory cytokines (Figure S8B) upon ATF6 complementation in PRSS1^Tg^/ATF6^-/-^ mice was also attenuated upon p53 inhibition. ATF6 overexpression in pancreas of caerulein-treated PRSS1^Tg^/p53^-/-^ mice using adenoviral delivery of ATF6 did not affect the attenuation in inflammatory cell infiltration, MPO activity, apoptotic cell numbers (Figure 6E), p53 and AIFM2 expression (Figure 6F), pancreatic edema, and serum amylase level (Figure S7C-E).

Western blotting (Figure 6C) and qRT-PCR (Figure 6D) analyses revealed that following p53 knockout in SAP tissues, the expression of AIFM2 was decreased but higher than control, while the expression of ATF6 did not change. Additionally, immunofluorescence analysis revealed that the increase in p53 expression upon SAP induction was attenuated upon ATF6 knockout (Figure S8C). These data indicate that p53 involves in acinar apoptosis and ATF6 regulates acinar apoptosis via p53-AIFM2 pathway during SAP development.

### ATF6 transcriptionally regulates p53 and AIFM2 expressions in acinar cells

**Figure 7:**
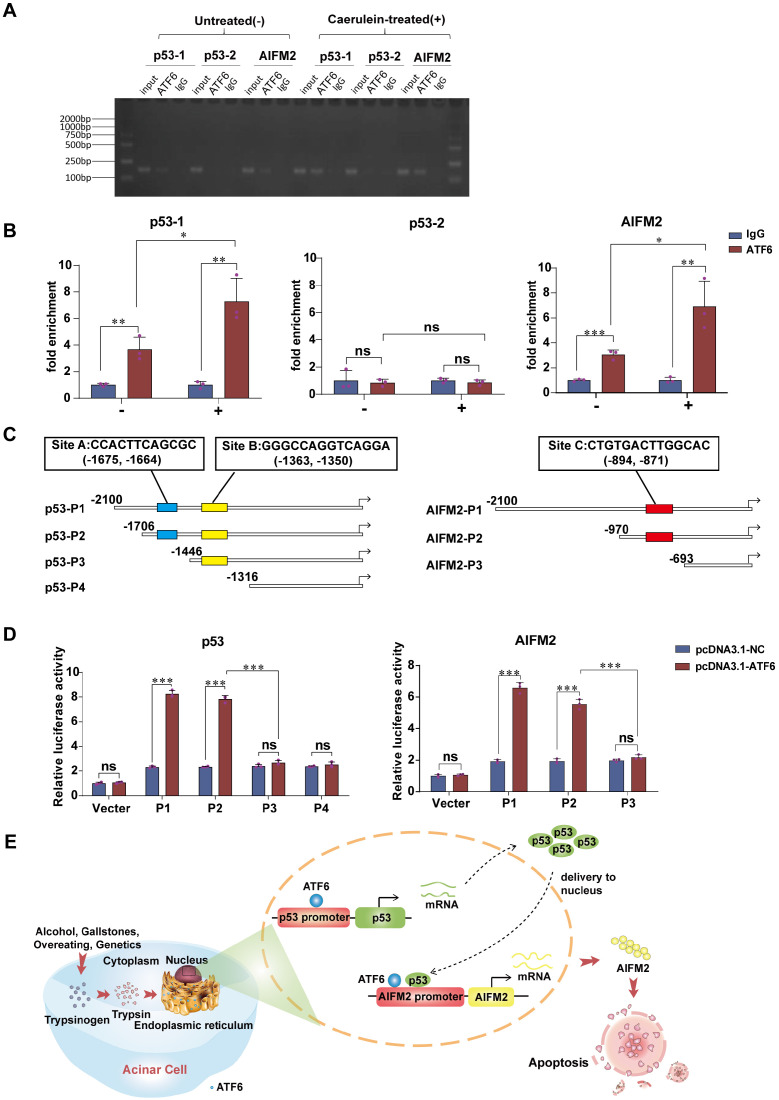
** ATF6 binds to the promoters of p53 and AIFM2 in acinar cells. (A**) Binding of ATF6 to p53 and AIFM2 promoter region in vitro by chromatin immunoprecipitation (ChIP) using anti-ATF6 or anti-IgG antibodies in untreated (-) or caerulein-treated (+) pancreatic acinar cells of PRSS1^Tg^ mice. **(B)** Input and immunoprecipitated DNA purified by ChIP were measured using qRT-PCR**. (C)** Schematic of deletion mutants from p53 and AIFM2 promoters. The predicted ATF6 binding sites were marked as Site A, B and C**. (D)** Luciferase reporter studies on the p53 and AIFM2 promoter in 293T cells. Cells were co-transfected with a series of mutants of p53 or AIFM2 promoter plasmid and either Atf6 overexpression plasmids or the control plasmids. Luciferase activities was measured 48h later. **(E)** A mechanistic figure for regulation of ATF6 on p53-AIFM2 pathway through apoptosis in SAP. The data are presented as the means ± SDs; ns, no significant difference; * p ≤ 0.05, ** p ≤ 0.01, *** p ≤ 0.001.

Due to its role of transcription factor, we speculate that ATF6 regulates the expression of AIFM2 by binding to the p53 and/or AIFM2 promoter in SAP. ChIP-qPCR (Figure 7A) was conducted to verify the prediction that there were two potential binding sites of ATF6 on the p53 promoter and one binding site on the AIFM2 promoter by Jaspar and hTFtarget. ChIP assay showed substantial increase in the binding of ATF6 to chromatin region of the p53-1 and AIFM2 promoter upon use of caerulein in pancreatic acinar cells, while p53-2 promoter exhibited no difference (Figure 7B), which indicated that ATF6 could bind to both the p53 promoter and the AIFM2 promoter, but ATF6 only bound to prediction site p53-1, not prediction site p53-2 in SAP. Based on the predicted binding sites of ATF6 to p53 and AIFM2 promoter (marked as Site A, B and C), four deletion mutants of p53 promoter (p53-P1, p53-P2, p53-P3, p53-P4) and three deletion mutants of AIFM2 promoter (AIFM2-P1, AIFM2-P2, AIFM2-P3) (Figure 7C) were respectively cloned into promoter-reporter vectors. Their activities in 293T cells were measured 48h after co-transfection by luciferase reporter system. Compared to p53-P2, p53-P3 and p53-P4, higher luciferase activities of p53-P1 suggested that ATF6 regulated p53 transcription by binding to the A site of the p53 promoter rather than the B site. ATF6-induced promoter activity of AIFM2-P2 was hardly declined in comparison with AIFM2-P1, indicating that ATF6 regulated the transcriptional activity of AIFM2 by binding to the C site of the AIFM2 promoter (Figure 7D). Together, using ChIP and luciferase assays, we conclude that ATF6 regulates the transcriptional activity of p53 by interacting with the p53 promoter through the consensus sites between -1706 and -1446, and ATF6 regulates the transcription of AIFM2 by interacting with the AIFM2 promoter at the consensus sites between -970 and -693 in SAP.

## Discussion

In the present study, we focused on understanding the importance of ATF6 for SAP development using a preclinical SAP mouse model which could mimic the “real world” performance of SAP. Our data identified ATF6 as a critical molecule in trypsin-induced acinar ER stress and apoptosis. Transcription factor ATF6 expression was elevated in pancreatic tissues of SAP and promoted acinar apoptosis. AIFM2, which is involved in the p53-induced apoptosis pathway, was upregulated by ATF6 in SAP. In turn, AIFM2 was downregulated when ATF6 was silenced, and this downregulation was accompanied with decreased apoptosis and inflammation. Moreover, ATF6 promoted p53 and AIFM2 transcription to induce acinar apoptosis in SAP (Figure 7E). These results provide the first indication that the AIFM2 regulates SAP with multiple organ injury via ATF6/p53 mediated apoptosis. Theoretically, ATF6 could be translated to a valuable therapeutic target to improve the dilemma of therapeutic limitations of SAP.

Current knowledge indicates that inflammation induces protein misfolding in the ER, which compromises cellular function and promotes apoptosis and the development of inflammatory diseases 10,11. To better understand the pathogenesis of SAP, we constructed a preclinical PRSS1^Tg^ mouse model that could mimic the development of SAP. Ultrastructural analysis of human pancreatic tissues and SAP model tissues from the pancreas, lung, liver, kidney, duodenum, heart, and spleen conducted using TEM revealed that the structures of cellular organelles, such as the ER, mitochondria and nucleus, changed significantly in multiple organ injury during the pathogenesis of SAP. Importantly, we observed significant increases in the destruction of ER and mitochondria, and in apoptosis (nucleus) in pancreatic tissues from both SAP model mice and patients. This manifestation suggests ER-mitochondrial-nuclear crosstalk involves and probably plays a role in SAP progression.

ATF6, a transmembrane-type glycoprotein on the ER membrane, functions as not only a UPR sensor/transducer, but also a transcription factor. When misfolded proteins accumulate in the ER, ATF6 transits to the Golgi apparatus, where it is processed to produce a cytosolic fragment that is the primary mediator of the adaptive response to ER protein misfolding 11,24. While the core function of ATF6 is to restore homeostasis, it can also induce apoptosis 25,26. In the present study, we found that ATF6 expression was elevated in pancreatic tissues from SAP patients and PRSS1^Tg^ mice and that it regulated acinar injury through apoptosis. The number of ATF6-linked gold beads was significantly higher in areas of ER rupture and nucleus, and mitochondrial swelling in SAP acinar cells compared to normal, as visualized by IEM. Interestingly, we found that ATF6-linked gold beads also surrounded the membrane of zymogen granules. This manifestation suggests an interaction between PRSS1 and ATF6. Importantly, we revealed that during SAP development, ATF6 promoted acinar apoptosis through binding p53 and AIFM2 promoter sites, which had never been reported. The above results confirm that ATF6 plays an important transcriptional role during SAP.

ER-mitochondrial interconnections form specific microdomains that play important roles in mitochondrial dynamics, inflammation, autophagy, and apoptosis 27. The physical and functional crosstalk between these two dynamic organelles emphasizes the outcome of altered ER-mitochondrial interconnections in SAP 28. Considering this interrelationship, we focused on identifying proteins that partner with ATF6 in roles related to mitochondrial functions and apoptosis. AIFM2 was identified as a functional ATF6-downregulating partner in SAP.

AIFM2, also named apoptosis-inducing factor-like mitochondrion-associated inducer of death (AMID) or PRG3, exhibits 90% similarity between mice and humans, is located on chromosome 10 in humans. It plays a role in mediating the p53-dependent apoptosis response and has been demonstrated to be an apoptosis-inducing factor-homologous mitochondrion-associated protein that induces caspase-independent apoptosis in humans and mice 22,23. AIFM2 expression is regulated by p53, which binds to p53-responsive elements in the AIFM2 promoter region 29. AIFM2 plays a key role in promoting cell proliferation, invasion, and migration 30,31. Also, AIFM2 was identified as a NADH oxidase, which regenerates NAD to promote glycolysis for thermogenesis 32. In human breast 33, gastric 34 and lung 35 cancer cells, apoptosis is mediated by the upregulation of AIFM2 expression and accumulation of AIFM2 in the mitochondria. In our SAP mouse model, AIFM2 accumulated mainly in the mitochondria. AIFM2 silencing significantly abated acinar apoptosis and injury; moreover, ATF6 knockdown downregulated AIFM2 expression, which was accompanied by reduced apoptosis and acinar injury in SAP. These results suggest a functional role for the ATF6-AIFM2 pathway in ER-mitochondrial-nuclear crosstalk in SAP. In addition to its role in apoptosis, AIFM2 was recently identified as a potent ferroptosis resistance factor (and is also called ferroptosis suppressor protein 1, FSP1), which functions as an oxidoreductase that reduces coenzyme Q10, generating a lipophilic radical-trapping antioxidant. FSP1 expression positively correlates with ferroptosis resistance across hundreds of cancer cell lines and FSP1 mediates resistance to ferroptosis in lung cancer both *in vivo* and *in vitro* 36,37. Therefore, AIFM2 may play different roles in different cell death pathways.

Under physiological conditions, p53 is expressed at low levels. Following DNA damage, p53 rapidly accumulates and becomes an active transcription factor for a set of downstream genes. Transcription of the human AMID gene is regulated by p53, and overexpression of AMID causes chromatin condensation at the nuclear periphery and apoptotic body formation 16,38, consistent with our results in SAP (Figure 6). Apoptosis induced by AMID is regulated by p53, is caspase-independent and is not affected by overexpression of the Bcl-2 protein 22,39. In addition to apoptotic functions, alterations in the mitochondrial membrane potential, mitochondrial ROS production and/or cytochrome C release can also result from p53-mediated transcriptional activation of mitochondrial proteins such as Noxa, p53, AIP1 and Bax 40. Our data also showed that p53 knockout in the PRSS1^Tg^ SAP mouse model significantly downregulated the expression of AIFM2 and alleviated acinar apoptosis and injury. Combined with our previous results (Figure 6, 7 and Figure S8), this experimental evidence demonstrates that the p53-AIFM2 pathway regulates acinar apoptosis and injury mediated by ATF6 in SAP, thus confirming the functional role of the ATF6-p53-AIFM2 pathway in SAP.

Coordination between the mitochondria and the nucleus is essential for maintaining cellular homeostasis and ensuring an effective response to stress. Under physiological or various cellular stress conditions, p53 can regulate diverse cellular processes, including DNA repair, the cell cycle, apoptosis, redox homeostasis and metabolism 41. In addition to its nuclear role, p53 can be localized to mitochondria, where it executes various transcription-independent functions 42. In our SAP mouse model, we found that p53 was localized mainly in the nucleus and cytoplasm (Figure 6A). Our ChIP and luciferase studies provide evidence that ATF6 binds to the p53 promoter and subsequently activates the p53-AIFM2 pathway. Moreover, the directly binding of ATF6 with the AIFM2 promoter synergistically regulates acinar apoptosis in SAP development (Figure 7). Thus, ATF6 activates the p53-AIFM2 pathway by binding to the p53 and AIFM2 promoter, leading to apoptosis.

In conclusion, our study sheds new light on a mechanism by which the p53-AIFM2 pathway regulates SAP with multiple organ injury via ATF6-mediated apoptosis, suggesting that ER-mitochondrial-nuclear crosstalk plays an important role in SAP development. These findings provide novel insights into ATF6-triggered signaling as a promising targeted therapy for pancreatitis and other common human diseases.

## Supplementary Material

Supplementary methods, figures and tables.

## Abbreviations

ATF6: activating transcription factor 6
AIFM2: apoptosis-inducing factor mitochondria-associated 2
SAP: severe acute pancreatitis
PRSS1: serine protease 1
PRSS1^Tg^: PRSS1 transgenic
qRT-PCR: quantitative real-time PCR
ChIP: chromatin immunoprecipitation
AP: acute pancreatitis
MOF: multiple organ failure
ER: endoplasmic reticulum
UPR: unfolded protein response
CP: chronic pancreatitis
PFT-α: pifithrin-α
DDA: data-dependent acquisition
DIA: data-independent acquisition
GO: Gene Ontology
COG: Clusters of Orthologous Groups
TEM: transmission electron microscopy
IEM: immunoelectron microscopy
PBS: phosphate-buffered saline
PB: phosphate buffer
PARP: poly ADP-ribose polymerase
MPO: myeloperoxidase
TUNEL: transferase-mediated d-UTP nick-end-labeling
ELISA: enzyme-linked immunosorbent assay
IL-1β: interleukin 1β
IL-6: interleukin 6
TNF-α: tumor necrosis factor α
ALT: alanine aminotransferase
AST: aspartate aminotransferase
CR: creatinine
CK-MB: creatine kinase-MB
LDH: lactate dehydrogenase
DEPs: differentially expressed proteins
AIF: apoptosis-inducing factor
PPI: protein-protein interaction
AMID: apoptosis-inducing factor-like mitochondrion-associated inducer of death
PRG3: p53-responsive gene 3
FSP1: ferroptosis suppressor protein 1
