# Supplementary material for: ATF6 aggravates acinar cell apoptosis and injury by regulating p53/AIFM2 transcription in Severe Acute Pancreatitis
Source: Theranostics. 2020 Jul 9;10(18):8298–314. doi: 10.7150/thno.46934 (PMC7381726; doi:10.7150/thno.46934)

Supplementary Methods

Transmission electron microscopy (TEM) and immunoelectron microscopy (IEM)

Ultrastructural examination was performed using a transmission electron microscope according to a previously described protocol [1]. Human pancreatic tissues (1 mm^3) and mice tissues (1 mm^3) from the heart, liver, spleen, lung, kidney, duodenum and pancreas were fixed with 2.5% glutaraldehyde at room temperature for 1 hour and then at 4°C overnight. After fixation, the samples were rinsed in phosphate-buffered saline (PBS) three times for 10 min each.

For immunogold staining, the experimental method has been adjusted, refering to the methods from the previous literatures [2,3]. Mice pancreatic tissues (1 mm^3) were fixed with a mixture of 4% paraformaldehyde and 0.1% glutaraldehyde dissolved in 0.1 mol/L phosphate buffer (PB) at 4°C overnight. Then, the specimens in PBS were sliced into 50-μm sections on a vibrating microtome (Lecia VT1200S).

The sections were fixed with a mixture of 4% paraformaldehyde and 0.1% glutaraldehyde dissolved in 0.1 mmol/L PB at 4°C for 2 hours and were then rinsed with 0.1 mol/L PB 3 times for 15 minutes each. Next, the sections were incubated with 0.05 mol/L glycine for 30 minutes and rinsed with 0.1 mol/L PB for 15 minutes. These steps were followed by incubation in 0.05% Triton X-100 dissolved in 0.1 mol/L PBS for 15 minutes, rinsing with 0.1 mol/L PB for 15 minutes, and sealing with 0.1% BSA-cTM for 30 minutes. Then, the sections were rinsed with incubation buffer (0.1 mol/L PBS+0.1% BSA-cTM) 2 times for 5 minutes each, incubated in incubation buffer containing the primary antibody at room temperature for 1 hour, and incubated with the primary antibody (diluted 1:200) at 4°C overnight. The next day, the sections were washed with 0.1 mol/L PBS+0.1% BSA-cTM 6 times for 10 minutes each. Then, the secondary antibody (Nanogold®-IgG goat anti-rabbit IgG (H+L), Nanoprobes) labeled

with gold colloid was applied at room temperature for 1 hour and incubated at 4°C overnight. Next, after successive washes with 0.1 mol/L PBS+0.1% BSA-cTM 6 times for 10 minutes each and PB 2 times for 10 minutes each, the sections were immobilized with 2.5% glutaraldehyde dissolved in 0.1 mol/L PB at room temperature for 2 hours. Then, the sections were washed successively with PB 3 times for 10 minutes each, double distilled water 6 times for 5 minutes each, and sodium citrate buffer 3 times for 5 minutes each. After silver enhancement for 6 minutes, the sections were washed with deionized water 6 times for 5 minutes each and rinsed with 0.1 mol/L PBS 3 times for 5 minutes each. The sections were stained with 1% osmium tetroxide at 4°C for 30 minutes, washed with double distilled water 3 times for 10 minutes each, and stained with 2% uranyl acetate for 30 minutes. After washing with 0.1 M PB twice for 10 minutes at 4°C, the sections was dehydrated in a graded water-ethanol series for 10 minutes each: 50%, 70%, 90%, followed by 3 × 100% ethanol at 4 °C. Replace 100% ethanol by ethanol-LR White (London Resin Company, England) 1:1, 1:2, 1:3 ; the time for each step is 1 hour, 2 hours, 3 hours at 4 °C. Then, infiltration with pure LR White resin at 4°C overnight. After the sections was transferred to a gelatin capsule containing LR white resin, polymerization by incubation at 65°C for 24 hours was performed. The resin-embedded sections were cut into ultrathin sections (70-80 nm thickness), and the sections were collected on nickel grids (Nisshin EM). Finally, the sections were analyzed with an electron microscope (Hitachi H-7500, Japan) operated at 60 kV.

Quantitative real-time PCR (qRT-PCR)

qRT-PCR was performed to measure mRNA expression in pancreatic tissues. Total RNA was extracted from samples using TRIzol reagent (Thermo Fisher Scientific, USA) according to the manufacturer’s protocol. Approximately 3.0 μg of total RNA was used for cDNA synthesis using the High Capacity cDNA Reverse Transcription Kit (Thermo

Fisher Scientific, USA) according to the manufacturer's protocol. Relative gene mRNA expression was evaluated by quantitative qRT-PCR using a QuantiFast SYBR Green PCR Kit (Qiagen, German) according to the manufacturer's instructions. GAPDH was used as an internal standard for normalization. At least three biological replicates were examined. The sequences of the primers used are listed in Supplemental Table S2.

Western blotting

Total protein was extracted from the mice pancreatic tissues using the Tissue or Cell Total Protein Extraction Kit (C510003; Sangon, Shanghai, China) following the manufacturer's instructions. After protein concentration determination, approximately 25 μg protein from each sample was denatured at 100°C for 5 minutes, separated by 12% SDS-PAGE, and transferred onto a PVDF membrane (Millipore). After blocking with 5% lipid-free milk solution, the membranes were incubated with primary antibodies at room temperature for 1-2 hours or 4°C overnight, washed three times with PBS solution for 5 minuters, and incubated with horseradish peroxidase-conjugated secondary antibodies for 2 hours. The immunocomplexes were finally developed with enhanced chemiluminescence solution (Thermo Fisher Scientific, USA). The protein abundance was evaluated by immunoblotting with at least three biological replicates.

Immunohistochemistry and Immunofluorescence assay

H&E staining and immunohistochemistry analysis of pancreatic tissue slides for ATF6, p53, AIFM2, Caspase-3, PARP and MPO were performed according to a previously described protocol [4]. To visualize ATF6, p53 and AIFM2 colocalization in pancreatic tissue, immunofluorescence assay was also performed.

Pancreatic tissues collected from patients and mice were fixed in 4% neutral phosphate-buffered formalin at 4 °C. Hematoxylin and eosin (H&E) staining was performed using 5-mm samples of pancreas by experienced pathologists at the

Pathological Department of Southern Medical University and histological evaluation was performed in a double-blinded manner. The immunohistochemistry assay based on the horseradish peroxidase system was performed using specific antibodies targeting ATF6 (Abcam; diluted 1: 100), p53 (Abcam; diluted 1: 100), AIFM2 (Biorbyt; diluted 1:100), Caspase-3 (Genetex; diluted 1:100), PARP (Genetex; diluted 1:100), and MPO (Abcam; diluted 1: 25). Positive staining of cells were observed and separately evaluated by two experienced pathologists. The ratio of positive cells in each sample was determined using the mean of 20 random microscopic fields.

To visualize ATF6, p53 and AIFM2 colocalization in pancreatic tissue, tissue slides were first incubated with antibodies against ATF6 (Abcam; diluted 1: 100), p53 (Abcam; diluted 1: 100), and AIFM2 (Biorbyt; diluted 1:100) at room temperature for 1hour followed by incubation with fluorescein isothiocyanate or phycoerythrin-conjugated secondary antibodies (Sangon, Shanghai, China). Tissues were observed using a fluorescent microscope.

Transferase-mediated d-UTP nick-end-labeling (TUNEL) assay

Terminal deoxynucleotidyl TUNEL was performed to detect apoptosis. Tissues were embedded in routine paraffin, de-waxed using dimethyl benzene, and hydrated using gradient ethanol. A TdT Frag DNA Fragmentation Imaging kit (Sigma Aldrich) was used to determine apoptosis according to the manufacturer’s instructions. Green staining in the nuclei of positive cells containing labeled DNA fragments indicated internucleosomal DNA cleavage. Three representative slides were chosen and five random visions of high field (× 200) were analyzed.

References:

1. Esrefoglu M, Gul M, Ates B, Selimoglu MA. Ultrastructural clues for the protective effect of melatonin against oxidative damage in cerulein-induced pancreatitis. J Pineal Res. 2006; 40: 92-7.

2. Reinshagen K. Autophagocytosis of the apical membrane in microvillus inclusion disease. Gut. 2002; 51: 514-21.
3. Tambasco De Oliveira P, Nanci A. Nanotexturing of titanium-based surfaces upregulates expression of bone sialoprotein and osteopontin by cultured osteogenic cells. Biomaterials. 2004; 25: 403-13.
4. Foygel K, Wang H, Machtaler S, Lutz AM, Chen R, Pysz M, et al. Detection of Pancreatic Ductal Adenocarcinoma in Mice by Ultrasound Imaging of Thymocyte Differentiation Antigen 1. Gastroenterology. 2013; 145: 885-94.

Supplementary Figures

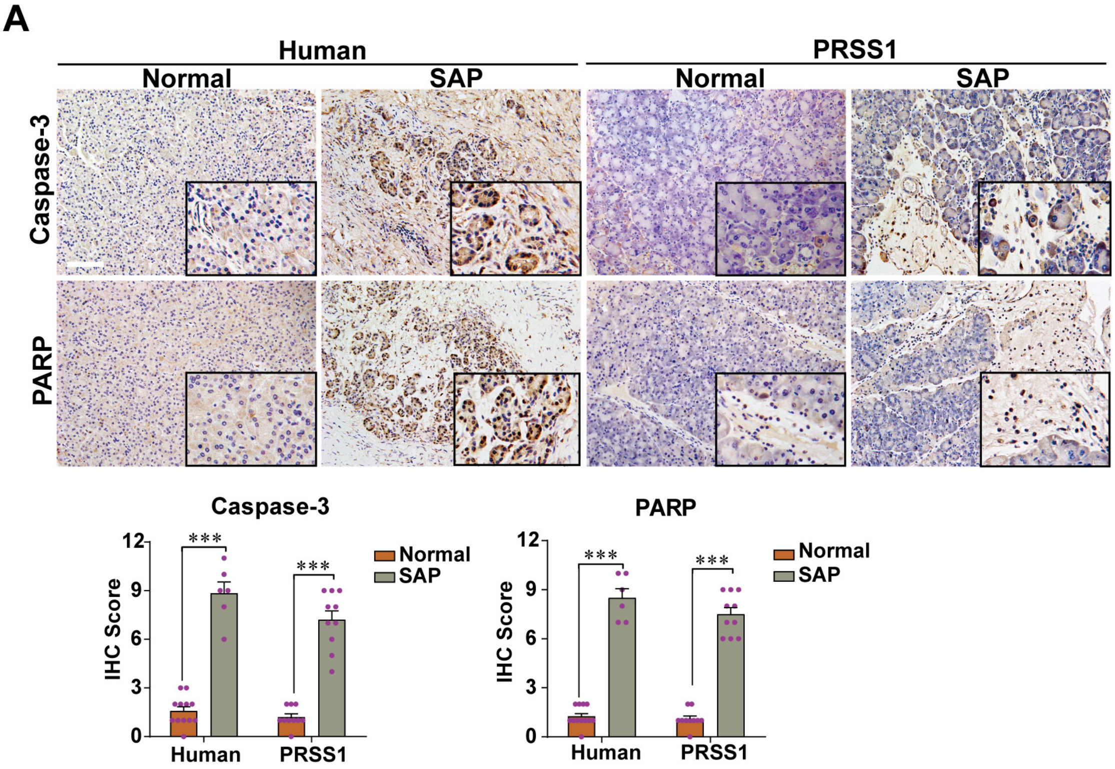

Figure S1. Expression of apoptotic markers is increased in SAP tissues from patients and PRSS1^{Tg} mice.

(A) The expression of the apoptosis-related proteins Caspase-3 and PARP in human and PRSS1^{Tg} mouse pancreatic tissues was analyzed by immunohistochemistry. The data are presented as the means ± SDs; * $p \leq 0.05$, ** $p \leq 0.01$, *** $p \leq 0.001$. Scale bars = 100 μm.

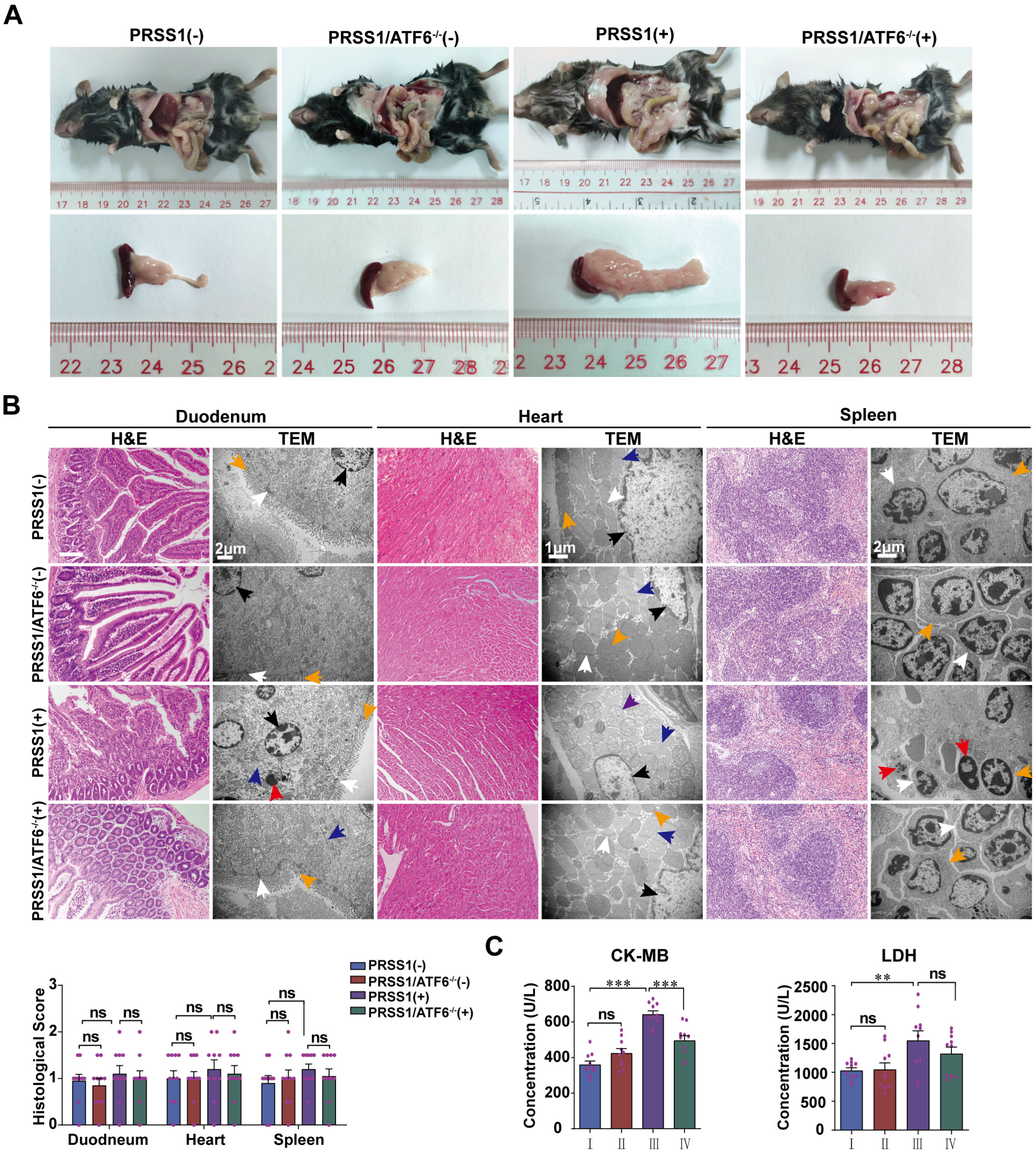

Figure S2. ATF6 promotes injury to other organs in the PRSS1^{Tg} SAP model. Morphological alteration **(A)** of mouse pancreas in caerulein-treated PRSS1^{Tg} mice and PRSS1^{Tg}/ATF6$^{-/-}$ mice. **(B)** Histological and microstructural evaluation of duodenum, heart, and spleen tissues collected from caerulein-treated PRSS1^{Tg} mice and PRSS1^{Tg}/ATF6$^{-/-}$ mice by H&E staining and TEM, respectively; black arrows (↑): cell nuclei; red arrows (↑): apoptotic bodies; purple arrows (↑): mitochondria; orange arrows (↑): microvilli (duodenum), intercalated discs (heart), B lymphocytes (spleen); white

arrows (↑): tight junctions (duodenum), Z-disk (heart), T lymphocytes (spleen); blue arrows (↑): gap junction (duodenum), myofibrils (heart). **(C)** Expression of heart-related CK-MB and LDH in serum from caerulein-treated PRSS1^{Tg} mice and PRSS1^{Tg}/ATF6$^{-/-}$ mice. (-), not treated with caerulein; (+), treated with caerulein. Group I, PRSS1^{Tg} (-); group II, PRSS1^{Tg}/ATF6$^{-/-}$ (-); group III, PRSS1^{Tg} (+); group IV, PRSS1^{Tg}/ATF6$^{-/-}$ (+). The data are presented as the means ± SDs; ns, no significant difference; * $p \leq 0.05$, ** $p \leq 0.01$, *** $p \leq 0.001$. Scale bars = 100 μm.

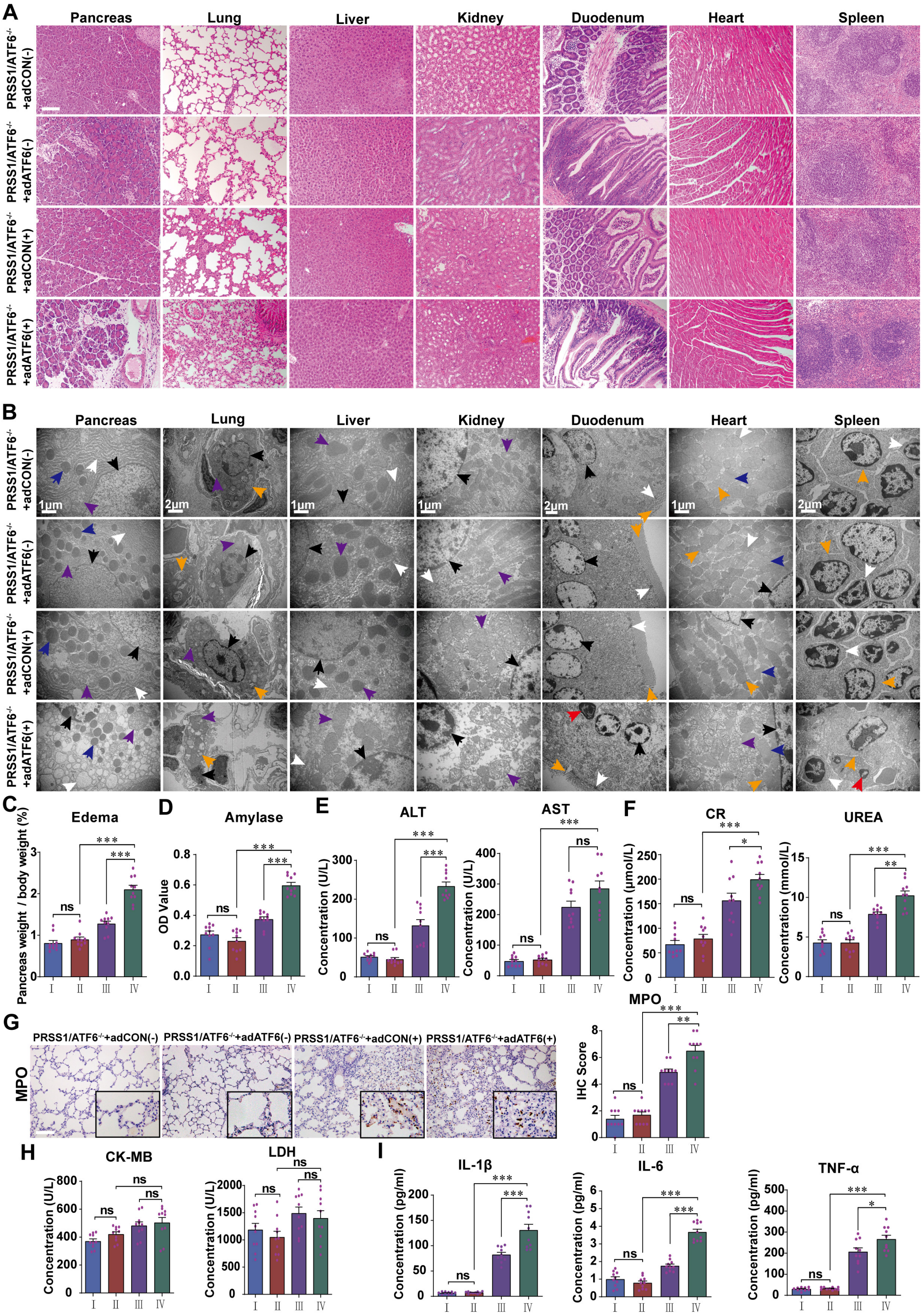

Figure S3. Overexpression of ATF6 reestablishes multiple organ injury in the PRSS1^{Tg} SAP model.

ATF6-expressing adenovirus (adATF6) for ATF6 overexpression or control adenovirus

(adCON) for the negative control were delivered to the pancreas of PRSS1^{Tg}/ATF6$^{-/-}$ mice. Representative images of H&E staining **(A)** and TEM **(B)** of pancreas, lung, liver, kidney, duodenum, heart, and spleen tissues in PRSS1^{Tg}/ATF6$^{-/-}$ mice; black arrows (↑): cell nuclei; purple arrows (↑): mitochondria; red arrows (↑): apoptotic bodies; white arrows (↑): endoplasmic reticula (pancreas, liver, kidney), tight junctions (duodenum), Z-disk (heart), T lymphocytes (spleen); blue arrows (↑): zymogen granules (pancreas), myofibrils (heart); orange arrows (↑): lamellar bodies (lung), microvilli (duodenum), intercalated discs (heart), B lymphocytes (spleen). Degree of edema **(C)** and serum amylase level **(D)** in PRSS1^{Tg}/ATF6$^{-/-}$ mice treated with adATF6 or adCON. The levels of liver-related ALT and AST **(E)** and kidney-related CR and urea **(F)** in serum from PRSS1^{Tg}/ATF6$^{-/-}$ mice treated with adATF6 or adCON. The immunohistochemistry for MPO in lung tissues was performed **(G)**. **(H)** Levels of heart-related CK-MB and LDH in serum from PRSS1^{Tg}/ATF6$^{-/-}$ mice treated with adATF6 or adCON. **(I)** Levels of IL-1β, IL-6, and TNF-α in serum were measured by ELISA from PRSS1^{Tg}/ATF6$^{-/-}$ mice treated with adATF6 or adCON. (-), not treated with caerulein; (+), treated with caerulein. Group I, PRSS1^{Tg}/ATF6$^{-/-}$+adCON (-); group II, PRSS1^{Tg}/ATF6$^{-/-}$+adATF6 (-); group III, PRSS1^{Tg}/ATF6$^{-/-}$+adCON (+); group IV, PRSS1^{Tg}/ATF6$^{-/-}$+adATF6 (+). The data are presented as the means ± SDs; ns, no significant difference; * $p \leq 0.05$, ** $p \leq 0.01$, *** $p \leq 0.001$. Scale bars = 100 μm.

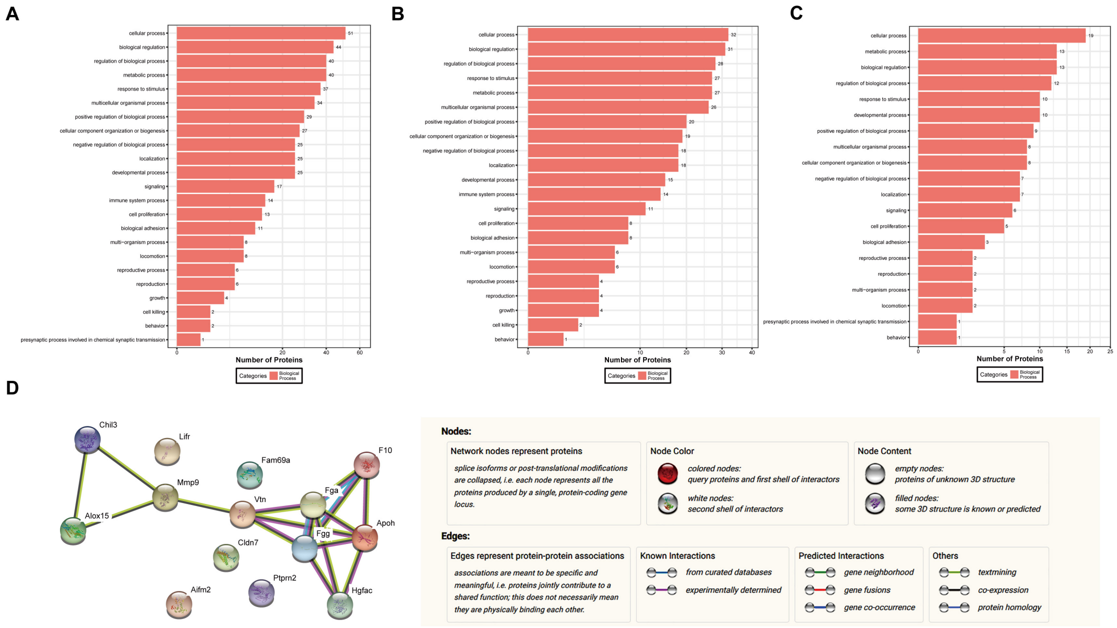

Figure S4. Proteomic analysis of differentially expressed proteins (DEPs).

(A) Gene Ontology (GO) functional annotation. The GO classification map shows the upregulated proteins **(B)** and downregulated proteins **(C)**. **(D)** The protein-protein interaction (PPI) network of 14 DEPs constructed using the STRING database.

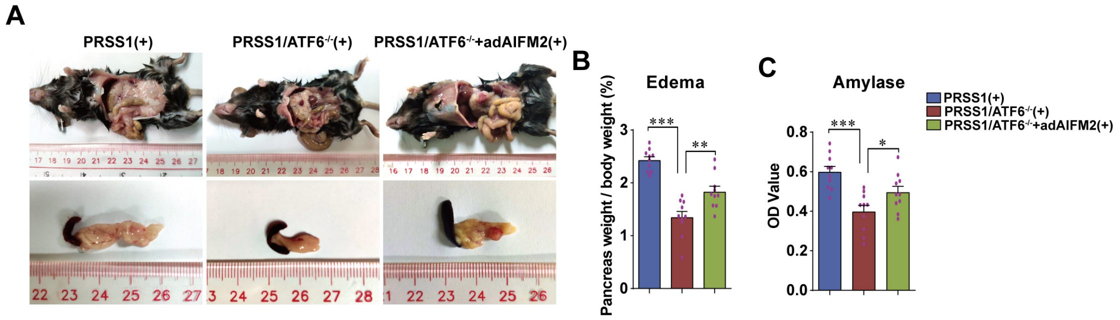

Figure S5. SAP performance is reestablished in PRSS1^{Tg}/ATF6$^{-/-}$ mice upon restoration of AIFM2 expression.

Morphological alterations in the pancreas **(A)**, degree of edema **(B)** and serum amylase level **(C)** in caerulein-treated PRSS1^{Tg} mice, PRSS1^{Tg}/ATF6$^{-/-}$ mice, and PRSS1^{Tg}/ATF6$^{-/-}$+adAIFM2 mice. The data are presented as the means ± SDs; * $p \leq 0.05$, ** $p \leq 0.01$, *** $p \leq 0.001$.

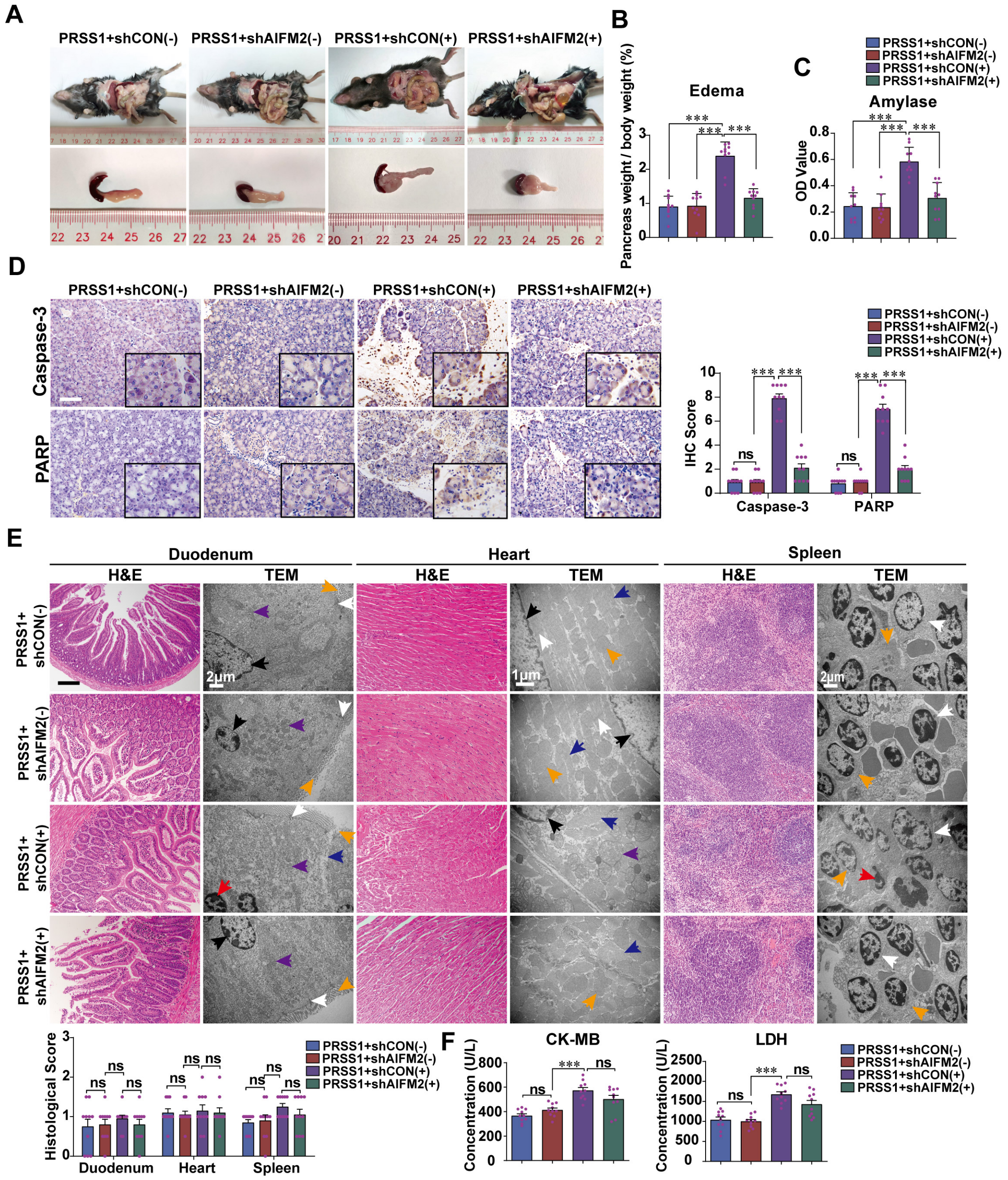

Figure S6. AIFM2 silencing ameliorates injury to other organs.

Morphological alteration of mouse pancreas **(A)**, degree of edema **(B)** and serum amylase level **(C)** in caerulein-treated PRSS1^{Tg} SAP model mice with shAIFM2-mediated AIFM2 silencing. **(D)** Immunohistochemical staining and scores for the apoptosis-related Caspase-3 and PARP proteins in pancreatic tissues from PRSS1^{Tg} SAP model mice with AIFM2 silencing. **(E)** Histological and microstructural

evaluation of duodenum, heart, and spleen tissues from caerulein-treated PRSS1^{Tg} SAP model mice with shAIFM2-mediated AIFM2 silencing; black arrows (↑): cell nuclei; red arrows (↑): apoptotic bodies; purple arrows (↑): mitochondria; orange arrows (↑): microvilli (duodenum), intercalated discs (heart), B lymphocytes (spleen); white arrows (↑): tight junctions (duodenum), Z-disk (heart), T lymphocytes (spleen); blue arrows (↑): gap junction (duodenum), myofibrils (heart). (F) Levels of CK-MB and LDH in serum from PRSS1^{Tg} mice treated with shAIFM2 or shCON. The data are presented as the means ± SDs; ns, no significant difference; * $p \leq 0.05$, ** $p \leq 0.01$, *** $p \leq 0.001$. Scale bars = 100 μm.

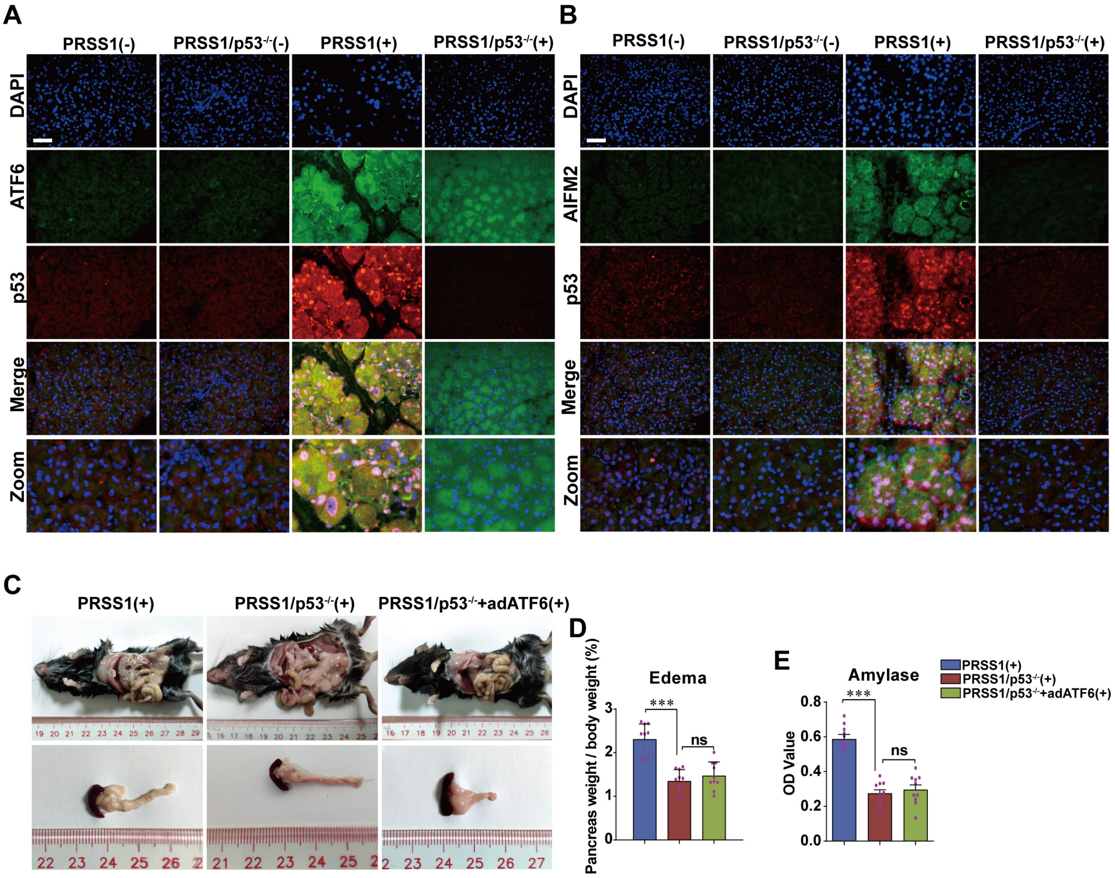

Figure S7. p53 knockout ameliorates acinar injury during SAP.

(A) The colocalization of ATF6 (green fluorescence) and p53 (red fluorescence) in pancreatic tissues from PRSS1^{Tg} and PRSS1^{Tg}/p53$^{-/-}$ mice was examined by immunofluorescent staining. **(B)** The colocalization of AIFM2 (green fluorescence) and p53 (red fluorescence) in pancreatic tissues from PRSS1^{Tg} and PRSS1^{Tg}/p53$^{-/-}$ mice was examined by immunofluorescent staining. Morphological alterations in the pancreas **(C)**, degree of edema **(D)** and serum amylase level **(E)** in caerulein-treated PRSS1^{Tg} mice, PRSS1^{Tg}/p53$^{-/-}$ mice, and PRSS1^{Tg}/p53$^{-/-}$+adATF6 mice. The data are presented as the means ± SDs; ns, no significant difference; * $p \leq 0.05$, ** $p \leq 0.01$, *** $p \leq 0.001$. Scale bars = 100 μm.

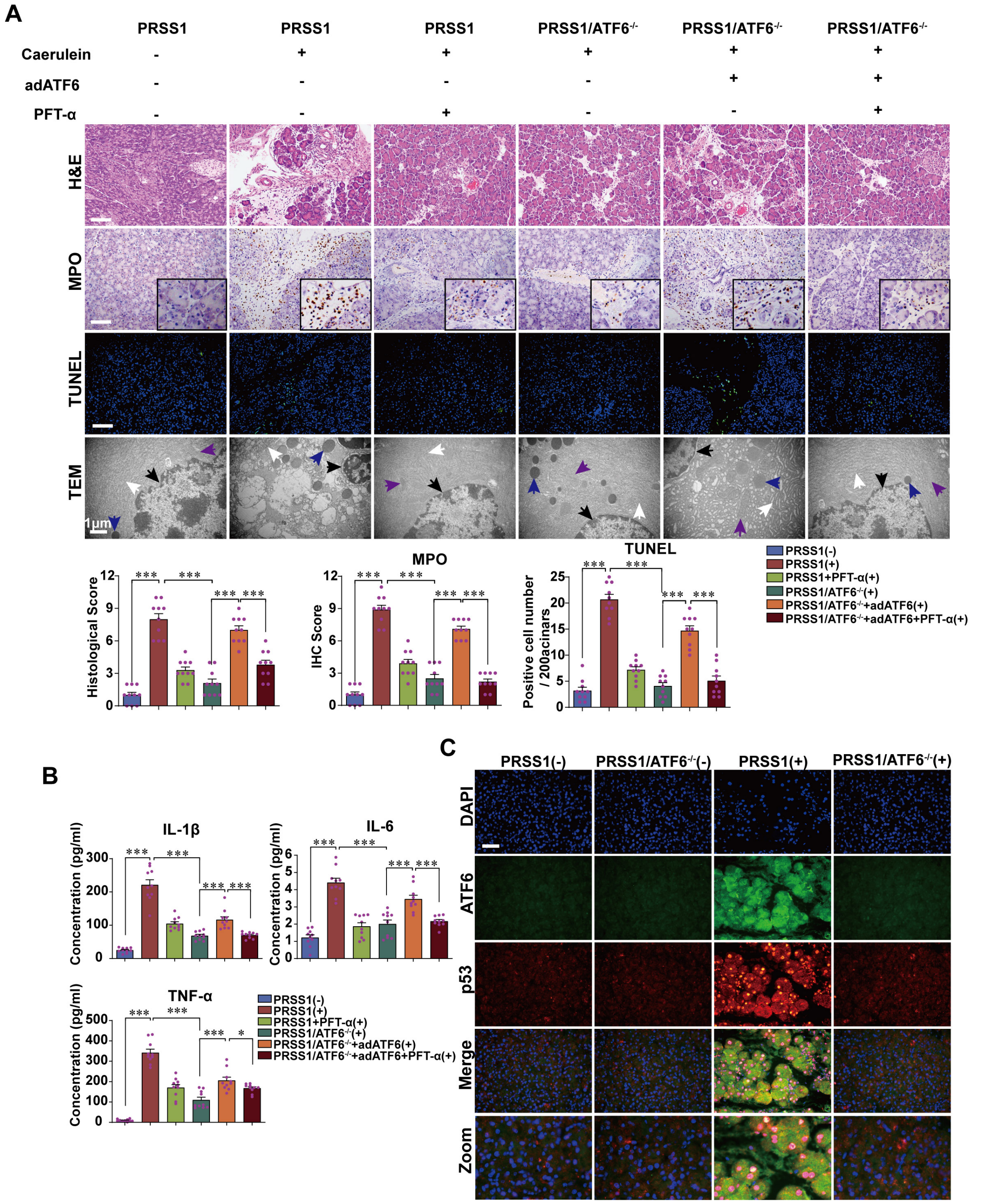

Figure S8. p53 is regulated by ATF6 during SAP.

(A) Untreated (-) or caerulein-treated (+) PRSS1^{Tg} or PRSS1^{Tg}/ATF6$^{-/-}$ mice were treated with PFT-α or saline. ATF6-expressing (adATF6) or control adenovirus (adCON) were delivered to the pancreas of PRSS1^{Tg}/ATF6$^{-/-}$ mice, followed by H&E staining, MPO immunohistochemistry, TUNEL, and TEM showing the pathological changes in

$PRSS1^{Tg}/ATF6^{-/-}$ mice and ATF6 deficiency in ATF6-rescued $PRSS1^{Tg}$ mice. **(B)** Expression of IL-1β, IL-6, and TNF-α in serum were measured by ELISA from ATF6-deficient $PRSS1^{Tg}$ mice and ATF6 deficiency in ATF6-rescued $PRSS1^{Tg}$ mice. **(C)** Immunofluorescent staining to examine the colocalization of ATF6 (green fluorescence) and p53 (red fluorescence) in pancreatic tissues from caerulein-treated $PRSS1^{Tg}/ATF6^{-/-}$ mice. The data are presented as the means ± SDs; * $p \leq 0.05$, ** $p \leq 0.01$, *** $p \leq 0.001$. Scale bars = 100 μm.

Supplementary Tables

Table S1. Clinical characteristic data of patients and normal controls

No.	age	Gender	Group	Complication
1	27	male	Normal	-
2	63	male	Normal	-
3	40	female	Normal	-
4	52	male	Normal	-
5	28	male	Normal	-
6	37	female	Normal	-
7	30	female	Normal	-
8	32	male	Normal	-
9	56	male	Normal	-
10	33	female	Normal	-
11	28	male	Normal	-
12	36	male	Normal	-
13	42	male	SAP	Lung failure
14	53	male	SAP	Lung and kidney failure
15	44	female	SAP	Kidney failure
16	49	female	SAP	Lung failure
17	55	male	SAP	Lung failure
18	47	male	SAP	Kidney failure

Table S2. The sequences of the primers used

Primer ID	Sequences (5'-3')
**PRSS1**	(F) GAGCGGATTTGAACGTTGTG
	(R) TACTTGAAGAGATTTGGCGG
**ATF6**	(F) CGCCTTTTAGTCCGGTTCTT
	(R) CCAGTTGGTAACAATGCCATGT
**P53**	(F) GGCAGACTTTTCGCCACAG
	(R) CAGGCACAAACACGAACCTC
**AIFM2**	(F) TTACAAGCCAGAGACTGACCAA
	(R) ACAAGGCCTGTCACTGAAGAG
**GAPDH**	(F) ATCATCAGCAATGCCTCCTG
	(R) ATGGACTGTGGTCATGAGTC

Table S3. The sequences of the primers for promoter region used

Primer ID	Sequences (5'-3')
**GAPDH promoter**	(F) CATGGGTGTGAACCATGAGA
	(R) GTCTTCTGGGTGGCAGTGAT
**Trp53 promoter**	(F1)GCTGTCGTTCTCACAGGTCC
	(R1)GAGTTGGAGTCAGGAGAAGC
	(F2) GGTCATCACCACCGCATG
	(R2) CTCTACAGAATGAAGACGTC
**AIFM2 promoter**	(F) CTATGTAGCTTCGAATGCTC
	(R) CTGAAGTCAGCCCTGAGCAAG

Table S4. Screened for related proteins

Gene names	Entry	Entry name	Protein names
Aifm2	Q8BUE4	FSP1_MOUSE	Ferroptosis suppressor protein 1
Alox15	P39654	LOX15_MOUSE	Arachidonate 15-lipoxygenase
Apoh	Q01339	APOH_MOUSE	Beta-2-glycoprotein 1
Chil3	O35744	CHIL3_MOUSE	Chitinase-like protein 3
Cldn7	Q9Z261	CLD7_MOUSE	Claudin-7
F10	O88947	FA10_MOUSE	Coagulation factor X
Fam69a	Q9D6I7	DIK1A_MOUSE	Divergent protein kinase domain 1A
Fga	E9PV24	FIBA_MOUSE	Fibrinogen alpha chain
Fgg	Q8VCM7	FIBG_MOUSE	Fibrinogen gamma chain
Hgfac	Q9R098	HGFA_MOUSE	Hepatocyte growth factor activator
Lifr	P42703	LIFR_MOUSE	Leukemia inhibitory factor receptor
Mmp9	P41245	MMP9_MOUSE	Matrix metalloproteinase-9
Ptprn2	P80560	PTPR2_MOUSE	Receptor-type tyrosine-protein phosphatase N2
Vtn	P29788	VTNC_MOUSE	Vitronectin

The images of western blotting

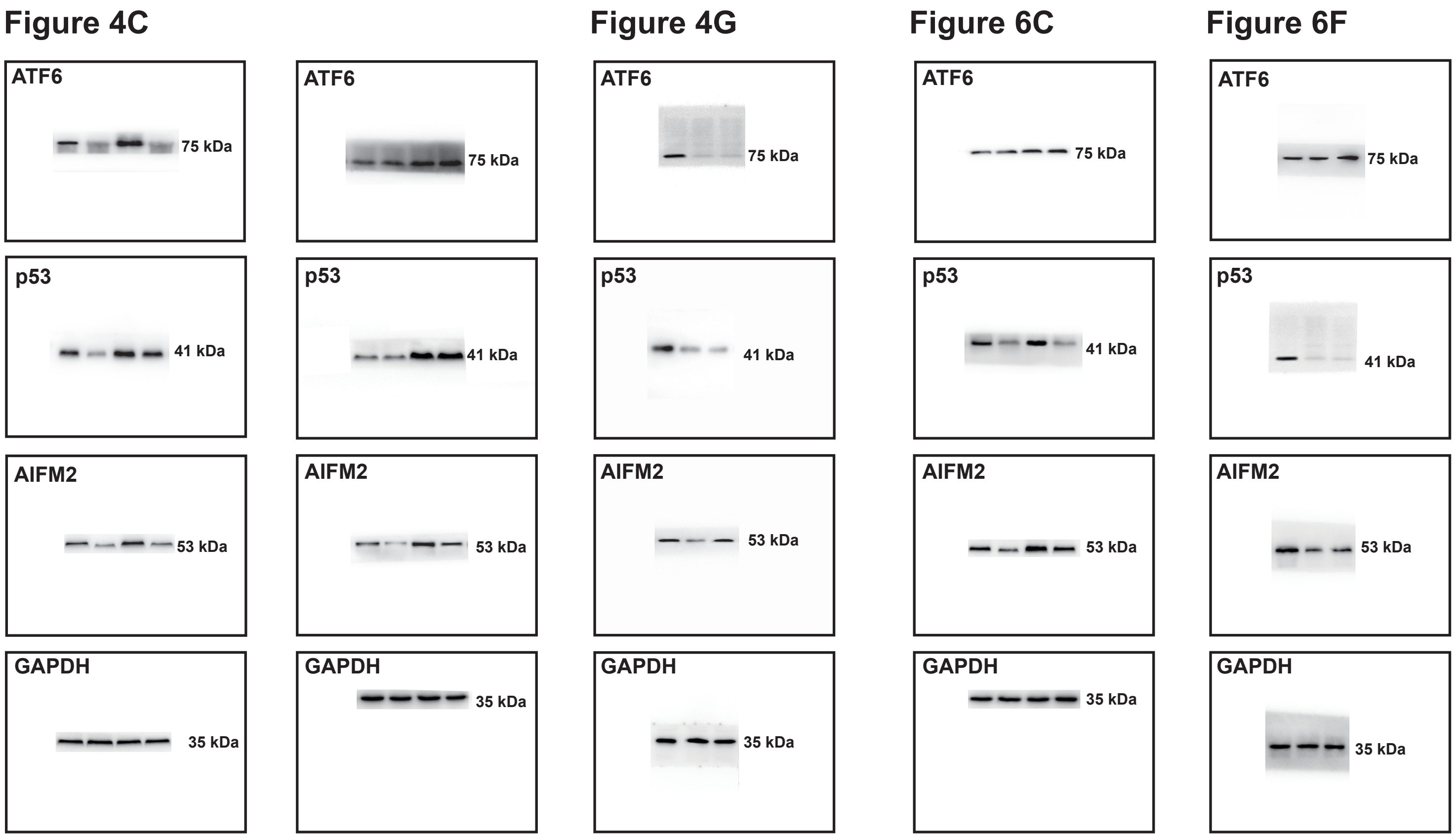

Supplement: Supplementary file 1 — Supplementary methods, figures and tables. [file thnov10p8298s1.pdf]
